# The DEAD-box RNA helicase PfDOZI imposes opposing actions on RNA metabolism in *Plasmodium falciparum*

**DOI:** 10.1038/s41467-024-48140-4

**Published:** 2024-05-03

**Authors:** Hui Min, Xiaoying Liang, Chengqi Wang, Junling Qin, Rachasak Boonhok, Azhar Muneer, Awtum M. Brashear, Xiaolian Li, Allen M. Minns, Swamy Rakesh Adapa, Rays H. Y. Jiang, Gang Ning, Yaming Cao, Scott E. Lindner, Jun Miao, Liwang Cui

**Affiliations:** 1 Department of Internal Medicine, Morsani College of Medicine, University of South Florida, 3720 Spectrum Boulevard, Tampa, FL 33612 USA; 2 Department of Immunology, College of Basic Medical Sciences, China Medical University, Shenyang, Liaoning China; 3 Center for Global Health and Infectious Diseases, Department of Global Health, College of Public Health, University of South Florida, Tampa, FL 33612 USA; 4 Department of Medical Technology, School of Allied Health Sciences, and Research Excellence Center for Innovation and Health Products (RECIHP), Walailak University, Nakhon Si Thammarat, 80160 Thailand; 5 Department of Biochemistry and Molecular Biology, Huck Center for Malaria Research, Pennsylvania State University, University Park, PA 16802 USA; 6 Electron Microscopy Facility, The Huck Institutes of the Life Sciences, Pennsylvania State University, University Park, PA 16802 USA

**Keywords:** Parasite biology, RNA decay, Parasite development

## Abstract

In malaria parasites, the regulation of mRNA translation, storage and degradation during development and life-stage transitions remains largely unknown. Here, we functionally characterized the DEAD-box RNA helicase PfDOZI in *P. falciparum*. Disruption of *pfdozi* enhanced asexual proliferation but reduced sexual commitment and impaired gametocyte development. By quantitative transcriptomics, we show that PfDOZI is involved in the regulation of invasion-related genes and sexual stage-specific genes during different developmental stages. PfDOZI predominantly participates in processing body-like mRNPs in schizonts but germ cell granule-like mRNPs in gametocytes to impose opposing actions of degradation and protection on different mRNA targets. We further show the formation of stress granule-like mRNPs during nutritional deprivation, highlighting an essential role of PfDOZI-associated mRNPs in stress response. We demonstrate that PfDOZI participates in distinct mRNPs to maintain mRNA homeostasis in response to life-stage transition and environmental changes by differentially executing post-transcriptional regulation on the target mRNAs.

## Abstract (web summary)

Min and colleagues study post-transcriptional regulation in the malaria parasite. They describe opposing actions of the DDX6-family RNA helicase complex on the decay and protection of specific mRNA targets during development and stress conditions.

## Introduction

Malaria control and elimination demand innovative technologies^1,2^, which would benefit tremendously from a better understanding of the biology of malaria parasites^3^. *Plasmodium* employs both global and transcript-specific translational regulation during development^4,5^. During the intraerythrocytic developmental cycle (IDC), ~30% of the transcripts experience a delay in translation^6–8^, suggesting a “just-in-time” translation mode. Post-transcriptional mechanisms are more appreciated during the life-stage transitions when the parasite forms (gametocytes and sporozoites) must remain quiescent for a relatively long period before transitioning to the next host. In these life stages, mRNAs needed for initiating subsequent development are stored in cytoplasmic messenger ribonucleoproteins (mRNPs) in a translationally repressed state and can be immediately mobilized for translation upon activation^4,9^. Despite ample evidence indicating coordinated post-transcriptional regulation during parasite development^10,11^, how it is mechanistically achieved is not clear.

In eukaryotic cells, the assembly of cytoplasmic membrane-less mRNPs into microscopically visible granules plays an important role in post-transcriptional regulation of gene expression^12^. Inside the cytoplasm, non-translating mRNAs can be packaged into different mRNP granules, such as the processing bodies (P-bodies, PBs), stress granules (SGs), and germ cell granules (GCGs), to regulate mRNA translation, decay, storage, and stress responses^12–15^. PBs include proteins involved in mRNA decay, including the deadenylase (CCR4/NOT complex), the decapping enzymes (DCP1/DCP2), and the decapping activator LSm1-7 complex. SGs are formed in response to stress conditions and contain translational initiation factors eIF3, eIF4A, E, and G, 40S ribosome subunits, and the polyA-binding protein (PABP)^12,13^. GCGs are identified in metazoan germ cells, known by many species-specific names such as P granules in *C. elegans*^16,17^. During development and under stress conditions, mRNAs may dynamically transit or cycle among polysomes, PBs, and SGs^12,13^. Malaria parasites encode many core proteins of mRNA metabolism^18^, but their roles in post-transcriptional regulation remain to be explored.

The DEAD-box protein DDX6 and its orthologs participate in mRNPs and are important for the storage, translational repression, and stability of mRNAs in somatic and germline cells^19–22^. It is highly conserved in evolution, and its orthologs are referred to as CGH-1 in *C. elegans*, Me31B in *Drosophila*, Xp54 in *Xenopus*, and DOZI in *Plasmodium berghei*^20,23^. Dhh1/Me31B represses translation and activates the decapping process^22,24,25^. DDX6 is expressed in different types of granules of the germ cells or embryos, where it associates with GCGs to repress translation and prevent degradation of maternal mRNAs^21,22^. In *P. berghei* female gametocytes, PbDOZI mRNPs resemble the SGs and the metazoan P granules and lack proteins for RNA decay^26^. The PbDOZI-CITH complex is associated with over 700 mRNAs and stabilizes them^27^. The human parasite *P. falciparum* has several distinctive features in sexual development, including a rather lengthy gametocytogenesis process and extended circulation time of mature gametocytes in the human host. However, it is unclear whether similar mechanisms are employed to preserve transcripts during the protracted quiescence.

In this study, we characterized the functions of *P. falciparum* DOZI (PfDOZI) and revealed its dynamic participation in distinct cytoplasmic granules during the asexual IDC and sexual development to deliver opposite effects on its diverse mRNA targets. We found that PfDOZI predominantly formed PB-like mRNPs in the asexual stages but GCG-like mRNPs in the gametocyte stage. *Pfdozi* disruption profoundly affected asexual development, sexual conversion, and gametocyte development, demonstrating the versatile roles of this RNA helicase in the stabilization, decay, and translational repression of different sets of mRNAs in *P. falciparum* in life-stage transition and stress response.

## Results

### PfDOZI localizes to cytoplasmic granules

**Fig. 1:**
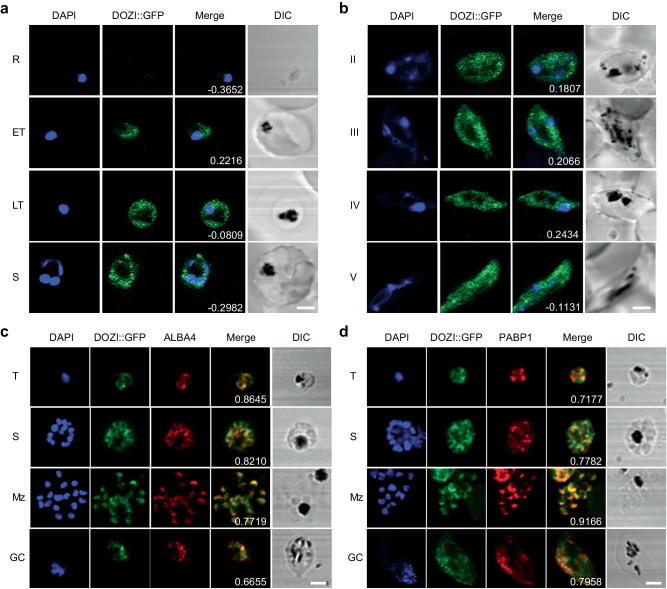
PfDOZI::GFP localizes to the cytosolic granules in asexual and sexual blood-stage parasites. **a**, **b** Representative live images of the PfDOZI::GFP parasites in asexual erythrocytic stages **a** and **b** stage II–V gametocytes. R, ring; ET, early trophozoite; LT, late trophozoite; S, schizont. Nuclei were counter-stained with DAPI. The numbers indicate the co-localization correlation coefficient between the GFP and DAPI fluorescence signals. Scale bar = 2 μm. **c**, **d** Co-localization of PfDOZI with Alba4 (**c**) and PABP1 (**d**) detected by IFA with the respective antibodies. T, trophozoite; S, schizont; Mz, merozoite; Gc, gametocyte. Nuclei were counter-stained with DAPI. The numbers indicate the co-localization correlation coefficient between the GFP and Alba4 or PABP1 fluorescence signals. DIC, differential interference contrast microscopy. Scale bar = 2 μm. All experiments in this figure were repeated three times independently with similar results.

Using the PfDOZI::GFP parasite line with the endogenous PfDOZI C-terminally tagged with a green fluorescent protein (GFP), we showed that PfDOZI was highly expressed in schizont and gametocyte stages (Fig. S1a–c). Live fluorescence microscopy showed that PfDOZI::GFP was localized in cytoplasmic puncta of the parasite during the IDC, with increasing numbers of foci in the late stage (Fig. 1a). While similar fluorescent foci were observed in all gametocyte stages (Fig. 1b), the intensity of the GFP signals differed among individual gametocytes. Flow cytometry analysis confirmed this observation, dividing gametocytes at a ratio of ~1:1.5 into two populations of low- and high-GFP fluorescent intensities (Fig. S2a). Indirect immunofluorescence assays (IFA) using antibodies against α-tubulin II, the male gametocytes marker^28^, identified preferential expression of PfDOZI in female gametocytes (Fig. S2b), consistent with the finding from the analysis of sex-specific proteomes^29,30^. Co-localization studies using two markers of mRNP granules, Alba4, and PABP1^10,26,31,32^, detected significant overlapping between PfDOZI::GFP and these two markers throughout development (Fig. 1c, d), suggesting the association of PfDOZI with mRNP granules marked by these RBPs.

### Disruption of *pfdozi* enhances parasite proliferation and erythrocyte invasion

**Fig. 2:**
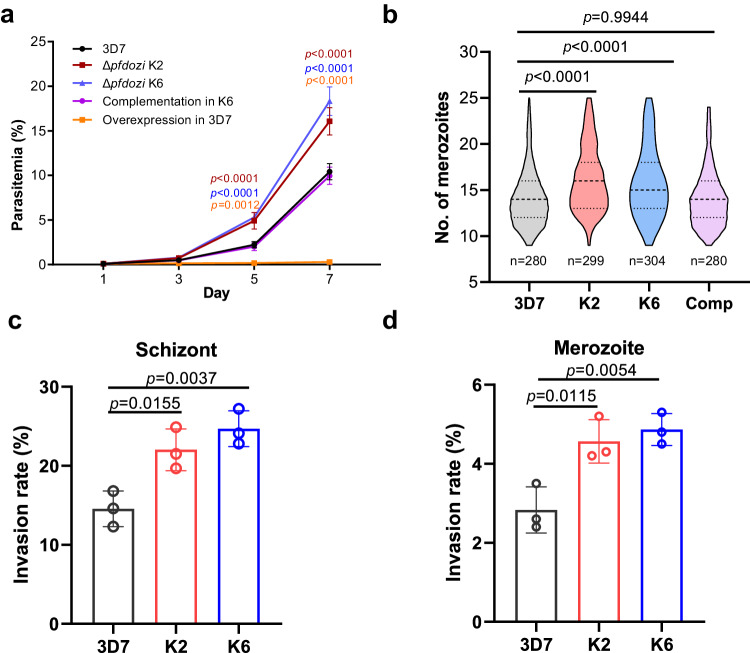
Growth phenotypes of the *Δpfdozi* parasite lines (K2 and K6) during the IDC. **a** Growth curves of the parasite lines. Cultures of all parasite lines were started at 0.1% parasitemia. Parasitemia was determined by flow cytometry every 48 h for three successive IDCs. Complementation and overexpression were performed by episomally expressing PfDOZI-tdTomato in *Δpfdozi* K6 and the WT 3D7, respectively. Data are shown as mean ± SD from three replicates. Two-way ANOVA following Dunnett’s multiple comparisons test was employed for comparison, *p*-values are indicated above the plots. **b** Violin plot depicting the distribution of the number of merozoites per mature schizont in 3D7 (*n* = 280), two *Δpfdozi* clones (K2, *n* = 299 and K6, *n* = 304), and the complementation (Comp) parasites (*n* = 280). The top dashline indicates the upper quartiles, the middle dashline indicates the median, and the bottom dashline indicates the lower quartiles. **c** Invasion rates of merozoites estimated following the natural rupture of mature schizonts. The number of input schizonts was adjusted by the average number of merozoites per schizont of each group, *n* = 3 for each group. **d** Invasion rates of merozoites estimated using purified merozoites. Error bars in all panels indicate the mean ± SD from three replicates. One-way ANOVA followed by Dunnett’s multiple comparisons test was applied for *p*-values in all panels. Source data are provided as a Source Data file.

PfDOZI shares a similar domain structure as other DDX6 family members, consisting of an ATP-binding domain and a C-terminal helicase domain (Fig. S3a). To study the functions of PfDOZI, we disrupted the *pfdozi* gene (*Δpfdozi*) by deleting the majority of the helicase domain (130 aa), which was verified by Southern blot, Western blot and RNA-seq (Fig. S3). Comparing parasite growth in two *Δpfdozi* lines (K2 and K6) and the wild-type (WT) 3D7 parasites during the IDC, we did not find noticeable differences in the gross morphology and stage progression between the *Δpfdozi* and 3D7 parasites (Fig. S4a, b). However, the *Δpfdozi* lines showed increased proliferation and produced significantly higher parasitemias than the 3D7 control (Fig. 2a, *p* < 0.01). Microscopic examination of mature schizonts revealed that the *Δpfdozi* lines produced significantly more merozoites per schizont (16.2 ± 3.5 for K2 and 15.8 ± 3.8 for K6) than the WT 3D7 (14.1 ± 3.1) (Fig. 2b, *p* < 0.0001). In addition, the *Δpfdozi* merozoites showed significantly higher invasion rates than the 3D7 control in erythrocyte invasion experiments using either isolated merozoites (K2 vs. WT, *p* = 0.0115, K6 vs. WT, *p* = 0.0054) or mature schizonts (K2 vs. WT, *p* = 0.0155, K6 vs. WT, *p* = 0.0037) (Fig. 2c, d). These results indicated that *pfdozi* disruption increased parasites’ proliferation rate mainly due to enhanced production and invasion efficiency of merozoites.

To confirm that phenotypic changes observed in the *Δpfdozi* lines were indeed due to *pfdozi* disruption, we performed complementation by episomally expressing PfDOZI in the *Δpfdozi* K6 clone (Fig. S5a). Expression of the tdTomato-tagged PfDOZI was verified by Western blot and live microscopy (Fig. S5b, c). Under these conditions, episomal expression of PfDOZI restored the growth and multiplication phenotypes of *Δpfdozi* to similar levels in the WT 3D7 (Fig. 2a, b). However, overexpression of PfDOZI-tdTomato in wild-type 3D7 was deleterious; transfected parasites were detected, but the parasitemia remained very low (Fig. 2a).

### Disruption of *pfdozi* reduces sexual conversion and impairs gametocyte development

**Fig. 3:**
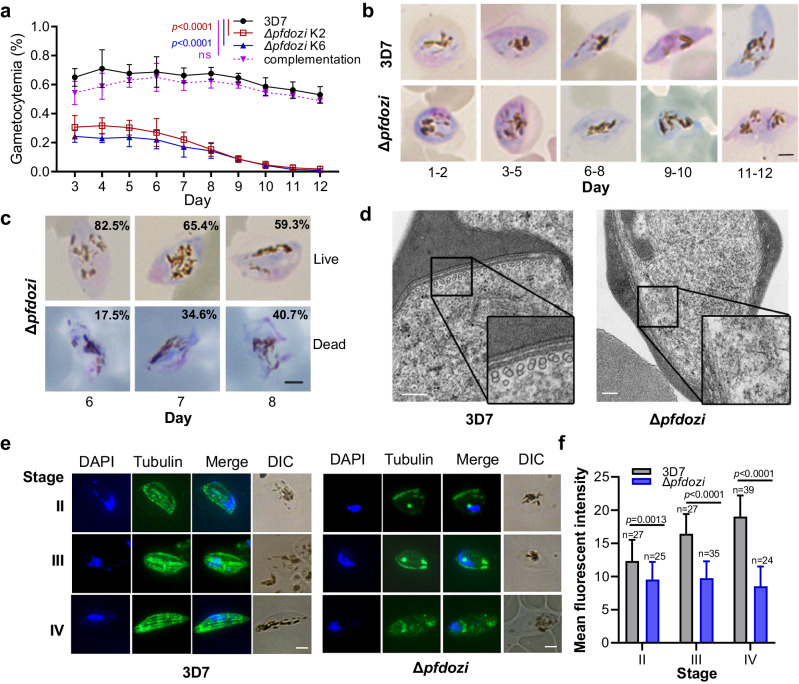
Gametocytogenesis and gametocyte morphology of the *Δpfdozi* parasite lines (K2 and K6). **a** Daily gametocytemia of two *Δpfdozi* clones (K2 and K6), 3D7 control, and complementation in clone K6 from day 3 through day 12 after induction. Gametocytemia was determined by counting Giemsa-stained gametocytes under a microscope. Data are shown as mean ± SD from three replicates. Two-way ANOVA following Dunnett’s multiple comparisons test was employed for comparison, *p*-values are indicated above the plots. **b** Representative images of Giemsa-stained gametocytes in 3D7 and *Δpfdozi* (K2) parasites at different days after induction. Scale bar = 2 μm. Similar results were obtained from three biological repeats. **c** Representative images of Giemsa-stained gametocytes illustrating the average percentage of live and dead gametocytes in the *Δpfdozi* line K2 on days 6–8. Scale bar = 2 μm. Similar results were obtained from three biological repeats. **d** Representative transmission electron microscope images showing the cytoskeleton structure of gametocytes in 3D7 and *Δpfdozi* parasites. The insets are the enlarged portions of the subpellicular structure showing the presence and absence of the microtubules in 3D7 and the *Δpfdozi* gametocytes, respectively. Scale bars = 200 nm. Similar results were obtained from two biological repeats. **e** Microtubule organizations in stage II (i), III (ii), and IV (iii) gametocytes of 3D7 (left panel) and *Δpfdozi* (right panel), Scale bars = 2 μm. Parasites were labeled with the anti-β-tubulin antibodies, and nuclei were counter-stained with DAPI. Similar results were obtained from two biological repeats. **f** Bar graph showing the mean β-tubulin immunofluorescence intensity in 3D7 and *Δpfdozi* gametocytes at different stages. Error bars indicate the mean ± SD. Two-tailed unpaired *t*-test, (i) *p* = 0.0013, degrees of freedom (df) = 50, (ii) *p* < 0.0001, df=60, (iii) *p* < 0.0001, df = 61. Source data are provided as a Source Data file.

Investigation of gametocyte development showed that the *Δpfdozi* lines had significantly lower initial gametocytemia than 3D7, suggesting a reduction in the sexual conversion rate (Fig. 3a, *p* < 0.01). Gametocyte development in the *Δpfdozi* lines appeared normal during the early stages (days 1–6) but stopped progressing beyond stage-III (Fig. 3b). Subsequently, dead gametocytes (aberrant cells) increased in culture on days 6–8, concomitant with a sharp decrease in gametocytemia (Fig. 3a–c). Notably, both male and female gametocytes were affected by the loss of PfDOZI despite PfDOZI being more abundantly expressed in females. These defects in sexual conversion and gametocyte development in the *Δpfdozi* K6 were almost completely rescued by episomally expressing PfDOZI-tdTomato (Fig. 3a).

The persistent, stage-III-like morphology of the developing gametocytes in *Δpfdozi* was reminiscent of the elongation defect observed in the knockdown of genes (e.g., PhIL1 and PIP1) encoding the inner membrane complexes (IMCs)^33^. This suggests that PfDOZI may have affected the cytoskeleton organization in the spindle- and banana-shaped stage VI and V gametocytes. To test this, ultrastructural analysis by transmission electron microscopy (TEM) showed a substantial reduction or disappearance of the microtubules and IMCs underneath the pellicles in *Δpfdozi* gametocytes (Fig. 3d, Fig. S6). Further, IFA with the anti-β-tubulin antibodies detected extensive disruption of the typical thread-like structure of the cytoskeleton (Fig. 3e) and a significant decrease in β-tubulin levels in *Δpfdozi* gametocytes (Fig. 3f, stage II, *p* = 0.0013, stage-III, *p* < 0.0001, stage IV, *p* < 0.0001). Thus, *pfdozi* disruption impaired sexual commitment and late-stage gametocyte development with defective cytoskeleton formation.

### *Pfdozi* disruption leads to an increased abundance of invasion-related genes in schizonts

**Fig. 4:**
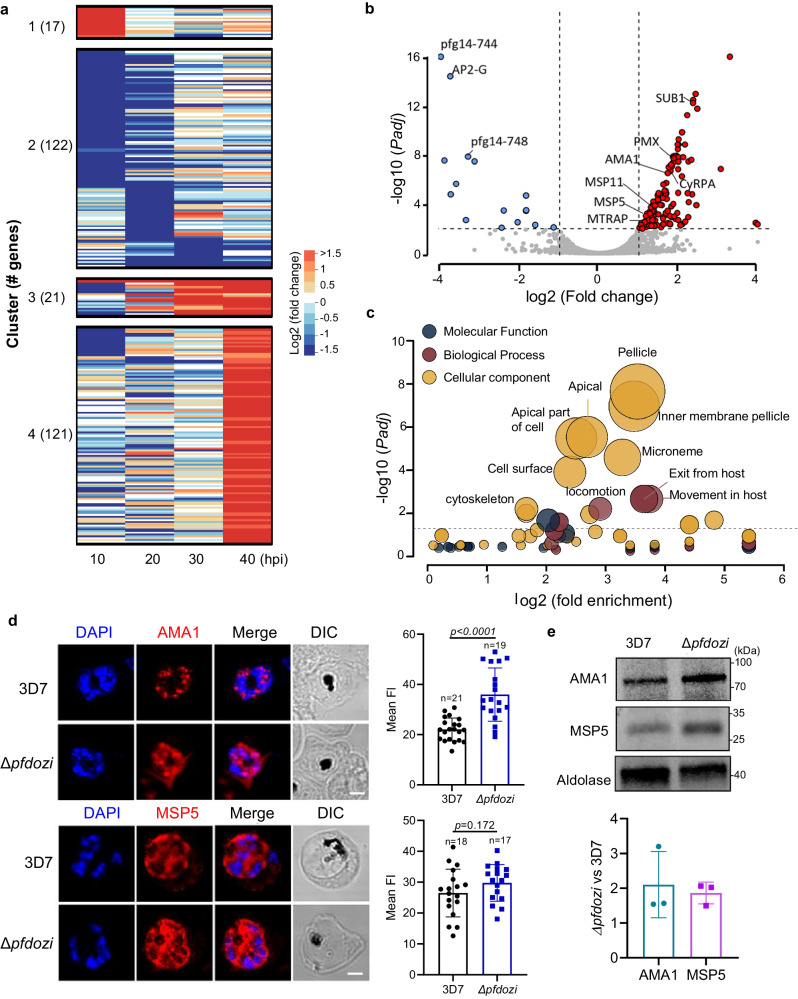
Transcriptomic analysis of gene expression during the IDC in the 3D7 and *Δpfdozi* parasites (K2). **a** The heat map depicting the *K*-mean clustering of differentially expressed genes in the *Δpfdozi* parasites at 10, 20, 30, and 40 h post-invasion (hpi) compared to the 3D7 parasites at the respective time points. **b** Volcano plot showing differential gene expression at the schizont stage (40 hpi) between *Δpfdozi* and WT 3D7 parasites. Each dot represents one gene and is displayed as log2 fold-change (*x*-axis) and the statistical significance of the association as -log_10_ (*Padj*) (*y*-axis). Transcripts with -log_10_ (*Padj*) above 16 were set as 16. Red dots indicate genes that are significantly increased in *Δpfdozi*, while blue dots are genes significantly decreased in *Δpfdozi*. Genes involved in RBC invasion and gametocytogenesis are labeled. **c** Bubble plot showing the gene ontology (GO) term analysis of the upregulated transcripts in the *Δpfdozi* parasite at 40 hpi. The three GO categories (cellular component, molecular function, and biological process) are shown in different colors. Only the GO terms with *p* < 0.05 are shown. The bubble size corresponds to the *p*-value of enrichment. Fold enrichment indicates the percentage of genes with a respective GO term in a group divided by the percentage of genes with this GO term in the whole genome. **d** Representative IFA images showing the PfAMA1 and PfMSP5 proteins in the 3D7 and *Δpfdozi* schizonts (left panel). Scale bar = 2 μm. Bar graphs in the right panel show the mean fluorescent intensity (FI) of PfAMA1 and PfMSP5 in the 3D7 and *Δpfdozi* schizonts at 40 hpi. *n* is the number of cells determined. Error bars indicate the mean ± SD (Two-tailed unpaired *t*-test, *p*-values are indicated above the plots). Similar results were obtained from two biological repeats. **e** Western blot analysis showing expression levels of PfAMA1 and PfMSP5 proteins in the 3D7 and *Δpfdozi* schizonts at 40 hpi (upper panel). PfAldolase was used as a loading control. The PfAMA1 and PfMSP5 protein bands were quantified by densitometry, normalized against PfAldolase, and the ratio between *Δpfdozi* and the WT 3D7 is shown in the bar graph (lower panel). Error bars indicate the mean ± SD from three replicates.

Because the DEAD-box helicases are involved in RNA metabolism, we wanted to determine whether *pfdozi* disruption impacted mRNA abundance in *P. falciparum*. RNA-seq analysis at four stages of the IDC identified 144 more and 137 less abundant transcripts in the *Δpfdozi* parasite compared to the WT (fold-change ≥ 2 and adjusted *P* (*Padj*) < 0.01, *var* gene transcripts excluded), which are grouped into four clusters by *K*-mean clustering (Fig. 4a, Fig. S7a, Supplementary Data 1: Table S1a–d). Clusters 1 and 4 include genes showing increased abundance at the ring and schizont stages, respectively, whereas Cluster 2 consists of all less abundant transcripts in the *Δpfdozi* parasite. For the 17 more and 73 less abundant transcripts at the ring stage in the *Δpfdozi* parasites (Fig. S7b), the Gene Ontology (GO) terms *pellicle structure*, *host cell*, and *long-chain fatty acid ligase activity* were enriched (Supplementary data 1: Table S1e). *Pfdozi* disruption minimally affected the trophozoites, with only 31 and 29 genes showing altered mRNA abundance at 20 and 30 h post-invasion (hpi), respectively (Fig. S7c, d). Among these 60 differentially expressed genes in trophozoites, an overwhelming majority (53) were less abundant in the *Δpfdozi* parasite (Fig. S7a). These genes with reduced mRNA abundance were enriched in multigene families encoding exported proteins. The most profound effect of *pfdozi* disruption on mRNA abundance was found at the schizont stage with 120 more and 11 less abundant transcripts. These genes with increased transcript levels normally had peak expression in schizonts (Fig. 4b), among which invasion-related genes and those associated with motor activity and cytoskeleton were highly enriched (Fig. 4b, c, Supplementary data 1: Table S1f), supporting the higher invasion efficiency of the *Δpfdozi* merozoites. The less abundant transcripts at 40 hpi include the master regulator of gametocytogenesis *ap2-g* and three gametocyte exported proteins (*gexp04, pfg14-744*, and *pfg14-748*), consistent with the reduced sexual conversion in *Δpfdozi*. The RNA-seq data for the gametocytogenesis-related genes were also verified by RT-qPCR analysis (Fig. S8). Notably, although the upstream gametocytogenesis regulator *gdv1* showed an increased level in *Δpfdozi* schizonts, the *ap2-g* transcript level was much lower, suggesting increased decay of *ap2-g* transcripts in the absence of PfDOZI.

To determine if the rising levels of invasion-related genes corresponded to increased protein levels, we quantified the microneme protein AMA1 and merozoite surface protein 5 (MSP5) in schizonts. IFA analysis showed that AMA1 protein, measured by the mean fluorescent intensity, was more abundant in *Δpfdozi* than WT parasites (*p* < 0.0001), whereas MSP5 protein did not show a pronounced increase in the *Δpfdozi* parasites (Fig. 4d, *p* = 0.172). Western blot confirmed that both AMA1 and MSP5 had an about 2-fold increase in *Δpfdozi* than WT schizonts (Fig. 4e). Taken together, these results indicated that the predominant effects of *pfdozi* disruption were destabilization of mRNAs encoding exported proteins in ring and trophozoite stages, and increased stability of invasion-related mRNAs in the schizont stage. The transcriptomic analysis also suggests that PfDOZI’s primary function switches from transcript stabilization to degradation during the IDC.

Since the altered mRNA abundance could result from changes in transcription or mRNA metabolism, we next examined whether the dysregulation of mRNAs in *Δpfdozi* was due to altered RNA decay rates. We focused our analysis on the schizont stage, given the most profound effect of *pfdozi* disruption in this stage. Highly synchronized schizonts at 40 h of both WT and *Δpfdozi* K6 parasite lines were treated with actinomycin D to block transcription. Transcript abundance was determined by RNA-seq analysis using parasite RNA obtained at 0–300 min after treatment (Supplementary data 1: Table S1g). Whereas we did not see a noticeable change in genome-wide RNA decay between the *Δpfdozi* and WT parasites, the 120 genes with increased abundance in *Δpfdozi* showed a significant right shift in the RNA decay curve compared to the WT, indicating more extended half-lives for these mRNAs (Fig. S9).

### *Pfdozi* disruption profoundly affects mRNA abundance in gametocytes

**Fig. 5:**
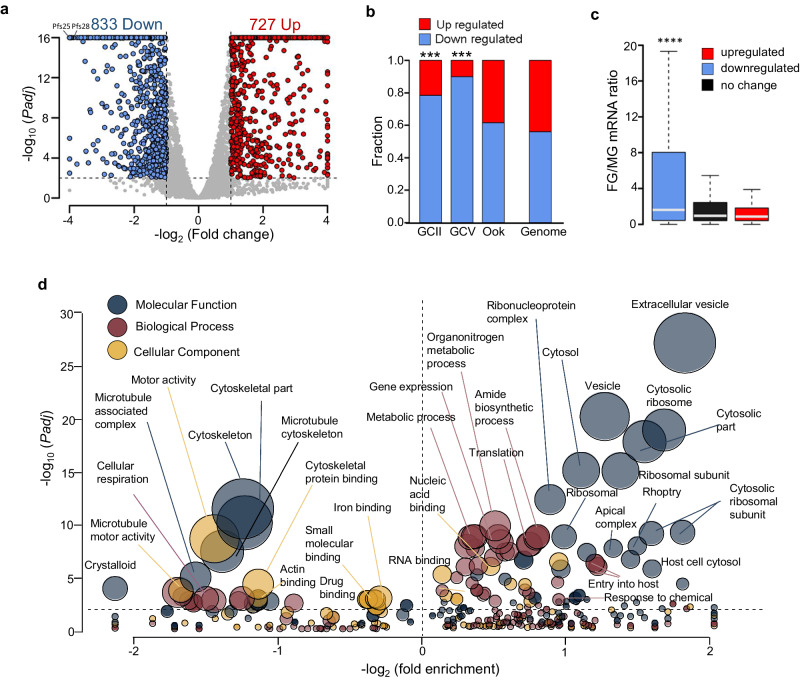
Transcriptomic analysis of gene expression in 3D7 and *Δpfdozi* gametocytes (K2). **a** Volcano plot showing the mRNA level difference between *Δpfdozi* and WT gametocytes. Each gene is plotted for its log2 fold-change (*x*-axis) and -log_10_*Padj* (*y*-axis). Transcripts with –log_10_ (*Padj*) above 16 were set as 16. Red and blue dots indicate genes that are significantly increased and decreased in *Δpfdozi*, respectively. **b** Bar plot depicting the fractions of genes with stage-specific expression in stage II gametocytes (GCII), stage V gametocytes (GCV), and ookinetes (Ook)^72^, which are upregulated (red) or downregulated (blue) in *Δpfdozi*. *** indicates that significantly larger numbers of genes downregulated upon *pfdozi* deletion are stage II- or III-specific genes (two-tailed Fisher’s test, *p* < 1e-5). Stage-specific genes are defined as those with >2-fold-change compared with all other stages in the RNA-seq analysis. The proportions of upregulated and downregulated genes in the whole genome are shown as the background. **c** Boxplot showing sex-specific expression of the genes that are downregulated (blue), upregulated (red), or unchanged (black) upon *pfdozi* deletion. Sex specificity is represented as the ratio of the transcript between female and male gametocytes (FG/MG). The downregulated genes in the *Δpfdozi* line had a significantly higher FG/MG ratio when compared with the other two groups (two-tailed Wilcoxon signed-rank test, *p* < 2.2e-16). The FG/MG ratio was based on the *P. falciparum* male and female gametocyte transcriptomes^29^. The boxplot illustrates the range between the lower and upper quartiles (Q1 and Q3) of the dataset, with the median (Q2) marked by a line within the box. Whiskers extend from the box to demonstrate the data’s spread, adhering to the following criteria: the upper whisker reaches the last data point below Q3 plus 1.5 times the interquartile range (IQR), while the lower whisker extends to the first data point above Q1 minus 1.5 times the IQR. Data points beyond the whiskers are plotted individually. **d** Bubble plot showing the enrichment of different GO terms in upregulated and downregulated genes in the *Δpfdozi* gametocytes. Only GO terms with *p* < 0.05 are shown. The bubble size corresponds to the *p*-value of enrichment. Fold enrichment indicates the percentage of genes with this GO term in the specific group divided by the percentage of genes with this GO term in the whole genome. The enriched GO terms in downregulated and upregulated genes are shown to the left and right of the vertical dotted line, respectively. The GO categories are shown in different colors. Details of the GO analysis are shown in Supplementary data: Table S2b and S2c.

Transcriptomic analysis of stage-III gametocytes revealed a more profound effect of *pfdozi* disruption, with 833 and 727 genes with less and more abundant transcripts in *Δpfdozi*, respectively (Fig. 5a, Supplementary Data 2: Table S2), suggesting equally important roles of DOZI complexes in mRNA preservation and degradation in mid-stage gametocytes. About 80% of those transcripts with reduced levels are gametocyte/ookinete-specific or -enriched transcripts (Fig. 5b), including 5 AP2-domain transcription factors, several 6-cysteine proteins, 8 CPW-WPC family proteins, 6 LCCL domain proteins, 6 secreted ookinete proteins, RBPs (Puf1 and Puf2), and many transcripts related to cytoskeleton and microtubule (Supplementary Data 2: Table S2a). Like in *pbdozi* deletion in *P. berghei*^26^, *pfs25* and *pfs28* encoding the major ookinete surface proteins were among the top genes with decreased transcript abundance, showing a respective 214- and 18-fold reduction. Consistent with preferential PfDOZI expression in female gametocytes, transcripts with reduced abundance in *Δpfdozi* possessed a high mRNA abundance ratio of female/male gametocytes^29^, indicating that transcripts with reduced abundance were female-biased (Fig. 5c). GO analysis showed that genes related to microtubule/cytoskeleton, cellular respiration, and crystalloid were significantly enriched among these genes, which agrees with the defective morphogenesis of the *Δpfdozi* gametocytes (Fig. 5d, Supplementary Data 2: Table S2b). Among the 727 genes with increased transcript levels, 11 RBPs, genes involved in translation (translation and ribosome), and exportome (exported proteins, host cell cytosol) were significantly enriched (Supplementary Data 2: Table S2c). Unexpectedly, nearly 11% of these genes showing increased transcript abundance encode the parasite egress and invasion machinery, which likely represents the remaining transcripts after the RBC invasion since their transcription is turned off in gametocytes^34^. Notably, the more or less abundant transcripts in *Δpfdozi* gametocytes accounted for 4.5% and 8.3% of total reads in the WT gametocyte transcriptome.

Given the more extensive overlaps of the less abundant genes in Δ*pfdozi* with those in Δ*pbdozi* and Δ*pbcith*^23,26^, and the PbDOZI and PbCITH target genes^27^ (Fig. S10), DOZI’s mRNA-preserving role appeared evolutionarily conserved. The more significant role of PfDOZI in female gametocytes is further reflected in the extensive overlap between the less abundant genes in Δ*pfdozi* and translationally repressed genes in *P. falciparum* female gametocytes^29^.

### RIP-seq analysis identifies the extensive PfDOZI regulons

**Fig. 6:**
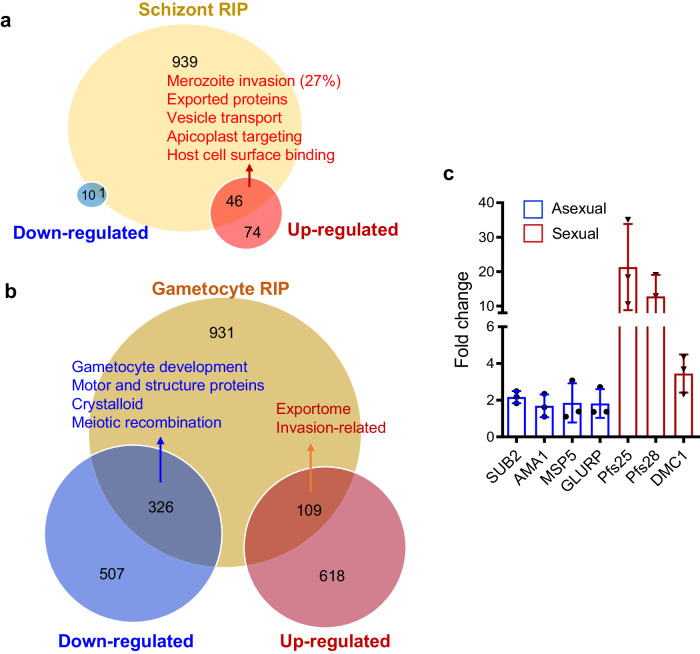
RIP-seq analysis of PfDOZI-associated mRNAs in *P. falciparum* schizonts and gametocytes. **a**, **b** Venn diagrams showing the overlap of transcripts between PfDOZI-associated mRNAs and differentially regulated genes in the *Δpfdozi* parasite at the schizont (**a**) and gametocyte (**b**) stage. The enriched pathways in each group were identified by the MPMP analysis. Blue, downregulated transcripts; Red, upregulated transcripts. **c** Real-time qRT-PCR confirmation of PfDOZI association with the selected transcripts. mRNAs immunoprecipitated using GFP-trap antibodies from the PfDOZI::GFP parasites at the schizont (blue) and gametocyte (red) stages were analyzed by qRT-PCR with primers specific to the indicated genes. Error bars indicate the mean ± SD of three independent experiments. All fold-change values are represented as the relative expression in comparison to the internal reference gene *PF3D7_0717700*. Source data are provided as a Source Data file.

To identify the mRNA regulons of PfDOZI in schizonts and gametocytes, we performed RNA immunoprecipitation and sequencing (RIP-seq) analysis using the PfDOZI::GFP parasites. We identified 986 and 1366 transcripts as potential targets of the PfDOZI complexes in schizonts and gametocytes, respectively (Fig. 6a, b; Supplementary Data 3: Table S3a–c). The Malaria Parasite Metabolic Pathways (MPMP) analysis of the RIP-seq data showed enrichment of gene sets in merozoite invasion, protein exports and host surface binding, and apicoplast proteins in the schizont stage, and AP2-domain transcription factors and genes in crystalloid formation in the gametocyte stage (Supplementary Data 3: Table S3d). The putative PfDOZI mRNA regulons overlapped significantly with genes whose expression was disturbed upon *pfdozi* disruption (*p* < 0.001, Fisher’s exact test). In particular, the RIP-seq enriched transcripts shared 47/131 (35.9%) and 435/1560 (27.9%) transcripts with altered abundance in *Δpfdozi* schizonts and gametocytes, respectively (Fig. 6a, b, Supplementary Data 3: Table S3e–g). When considering the putative DOZI-bound transcripts only, 46 and 1 gene had increased and decreased transcript abundance in *Δpfdozi* schizonts, while 109 and 326 transcripts partitioned into more and less abundant transcripts in *Δpfdozi* gametocytes (Fig. 6a, b). For these shared transcripts in schizonts, genes involved in RBC invasion, host cell remodeling, and apicoplast targeting are enriched (Fig. 6a). In gametocytes, genes involved in the parasite exportome and RBC invasion were enriched among the shared genes with increased abundance in *Δpfdozi*, while genes destined for gametocyte development, motor and structure functions, crystalloid formation, and meiotic recombination were enriched among those shared with the less abundant genes in *Δpfdozi* (Supplementary Data 3: Table S3e–g). A cross-species comparison of the RIP results showed that PfDOZI had 146 and 166 common mRNA targets with PbDOZI and PbCITH in gametocytes^27^. Further, RT-qPCR analysis was performed to verify the mRNA targets of PfDOZI in schizonts and gametocytes, respectively (Fig. 6c). Consistently, *p25* and *p28* were enriched in the PfDOZI::GFP gametocytes as shown in PbDOZI^23^.

### PfDOZI is associated with different mRNPs in schizonts and gametocytes

**Fig. 7:**
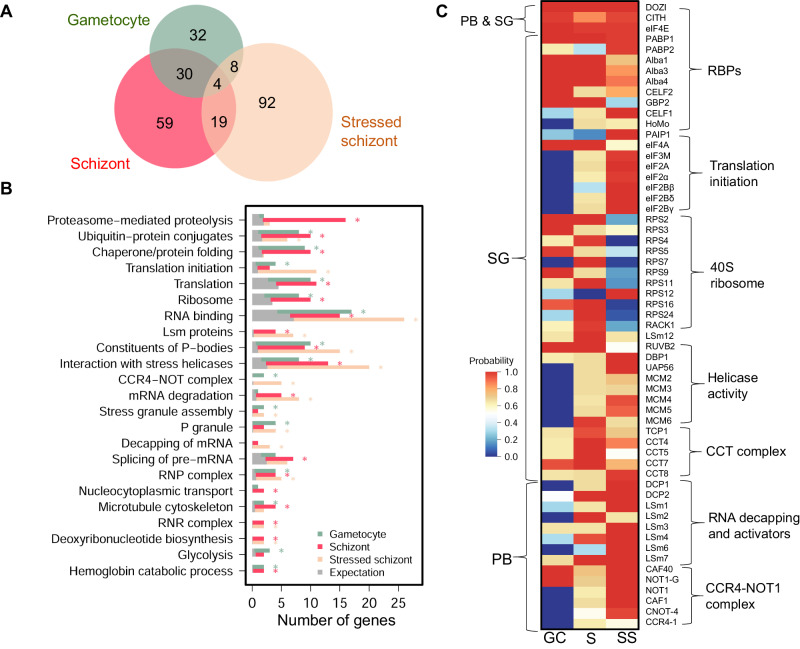
PfDOZI interactomes in different developmental stages and under different growth conditions. **A** Venn diagram depicting the overlap of identified proteins with an average probability of ≥0.9 and FDR below 1.0% from the PfDOZI interactome analysis. PfDOZI was immunoprecipitated from the lysates of schizonts, gametocytes, and stressed schizonts (under nutrient starvation) under native conditions, and the precipitated proteins were identified by LC-MS/MS. **B** Bar graph showing the number of genes in individual pathways significantly enriched in the PfDOZI interactomes from gametocytes (blue), schizonts (red), and stressed schizonts (pink), respectively. The gray bars indicate the expected number of genes in each term, calculated as the total number of identified genes multiplied by the fraction of genes in the query function term in the whole genome. The asterisks indicate significant enrichment of the pathway in the PfDOZI interactome at the specified stage. **C** Heat map of selected proteins in the PfDOZI interactomes in gametocytes (GC), schizonts (S), and stressed schizonts (SS) based on the average probabilities. The proteins were grouped based on their putative functions. Only those known to be associated with P-body (PB) and stress granule (SG) in other organisms are included.

Since the DDX6 RNA helicase is a component of various mRNPs in somatic cells (PBs and SGs) and germ cells (e.g., P granules) to carry out an array of functions in mRNA metabolism^12,13,21,35^, we hypothesized that PfDOZI might also be associated with different mRNPs to impose divergent actions on target mRNAs. To address this question, we performed immunoprecipitation (IP) using PfDOZI::GFP schizonts and purified gametocytes (day 5) with the GFP-trap beads under native conditions. We identified 112 PfDOZI-associated proteins in schizonts, including mRNA decay-related proteins typically found in PBs (the decapping enzymes DCP2 and DCP1, the decapping activators LSm1-4 and LSm7, and the deadenylase complex constituents NOT1, CAF1, and CAF40) and SGs [PABP1, G-strand binding protein 2–GBP2^36^, eIF4A and eIF4E, Alba 1, 3, and 4, components of the 40 S ribosome (9 ribosomal proteins and RACK1 kinase), LSm12, CELF1, and CELF2], suggesting the formation of both PB-like and SG-like mRNPs in schizonts (Fig. 7A, Supplementary Data 4: Table S4a-h). It is noteworthy that the mRNA decay-related proteins (DCP2 and LSm2/4/7) were relatively more abundant in schizonts than the core SG components such as eIF4E and CITH (LSm14). MPMP analysis identified the enrichment of PB-related categories (constituents of PB, cytoplasmic PB assembly, LSm proteins and RNA processing, mRNA degradation), whereas the SG-related categories were not enriched, suggesting a more prominent role of the PfDOZI complex in mRNA decay (Fig. 7B, Supplementary Data 4: Table S4b).

We identified 74 PfDOZI-associated proteins in gametocytes (Fig. 7, Supplementary data 4: Table S4c); 34 were shared with those identified in schizonts, including proteins typically found in PBs (CAF40 and NOT1) and SGs (PABP1, eIF4E, eIF4A, and Alba1/3/4) (Fig. 7A, Supplementary Data 4: Table S4h). Of the components previously identified in the DOZI, CITH, and Alba4 complexes in the rodent parasites *P. berghei* and *P. yoelii*^10,26^, 13 were identified in the PfDOZI complexes, including PfDOZI, eIF4E, PABP1, CITH, Alba1/3/4, CELF2/HoBo and enolase (Fig. 7C), whereas HoMu, Alba2, and phosphoglycerate mutase were not detected or did not pass the selection criteria. Compared to schizonts, the most distinct feature of the PfDOZI interactome in gametocytes is the enrichment of the SG- or GCG-related categories (P granule, stress granule assembly, and cytoplasmic stress granule), glycolysis (enolase and 6-phosphofructokinase), and CCR4-NOT complex (CAF40 and NOT1-G). Besides, the PfDOZI gametocyte interactome included seven additional RBPs. Except for lacking germ plasm proteins specific for metazoans, the gametocyte DOZI complex resembled the GCGs in *Drosophila* ovaries^37^, which is also consistent with the formation of P granule (*C. elegans* GCGs)-like mRNPs in gametocytes of the rodent parasites^10,26^.

The above interactome studies suggested dynamic changes in protein and mRNA compositions of PfDOZI mRNPs during *P. falciparum* development. Since malaria parasites are exposed to various stress conditions in their life history, we wanted to determine whether stress would alter the organization of mRNPs. After exposing PfDOZI::GFP to nutrient stress, we performed targeted proteomics of the schizont stage and identified 123 proteins (Supplementary Data 4: Table S4e). Compared with the normal culture conditions, nutrient starvation drastically changed the PfDOZI interactome, with only 23 proteins preserved between the two culture conditions (Fig. 7A, Supplementary Data 4: Table S4g). Particularly, several GO categories significantly enriched in normal schizonts (proteasome, chaperone and protein folding, ribosome, nucleocytoplasmic transport, and microtubule cytoskeleton organization) were not enriched in the stressed schizonts (Fig. 7B). Although the PB-related categories remained enriched, stressed schizonts had significant enrichment of mRNA degradation (CCR4-NOT complex, decapping) and the SG-related terms (stress granule assembly and P granule). The GO terms translation initiation, negative regulation of translation, and eIF2B complex were also enriched (Fig. 7B, Supplementary Data 4: Table S4f). In addition, the PfDOZI interactome in stressed schizonts also contained a plethora of ATP-dependent helicases and protein remodelers (chaperones and the chaperonin-containing T complex–CCT complex) (Fig. 7C). These ATP-dependent cellular machines are conserved constituents of the yeast and mammalian SGs, playing divergent roles in SG assembly^38^. Further, the 40S ribosomes were depleted under nutrient stress (Fig. 7C), reminiscent of the SGs formed under chronic nutrient starvation in mammalian cells^39^. These changes in PfDOZI-associated proteins in response to nutrient stress suggest the formation of SG-like granules.

The PfDOZI interactomes between stressed schizonts and gametocytes only shared 12 proteins, indicating major differences in the mRNP granules (Fig. 7A, B, Supplementary Data 4: Table S4g). The gametocyte DOZI interatomes included PABP1, CITH, eIF4A, eIF4E, GBP2, Alba1/3/4, and 40 S ribosomal proteins, resembling the GCGs in metazoans. In contrast, the SG components in the stressed schizonts contained PfDOZI, PABP2, CITH, PAIP1 (PABP-interacting protein 1), eIF2B complex, and eIF3M, but were deficient in eIF4A, the Alba proteins, GBP2, and the 40S ribosomal proteins (Supplementary Data 4, Table S4h). These differences suggest that gametocytes and schizonts may employ different mechanisms involving divergent PfDOZI complexes to regulate their target mRNAs.

Since the DOZI homolog is present in PBs, SGs, and GCGs in model eukaryotes, we wanted to determine whether it also partitions into different mRNPs in *P. falciparum*. To identify mRNP sub-complexes, we selected DCP2 and GBP2 as potential PB and SG markers, respectively. In model organisms, DCP2 is a constitutive PB component^40^, while GBP2 accumulates in SGs under stress conditions^41^. We tagged the endogenous loci of DCP2 and GBP2 with GFP at their C termini, and correct tagging was confirmed by integration-specific PCR and Western blots (Fig. S11). Protein pulldown and proteomic analysis of PfDCP2::GFP parasites at the schizont stage identified 34 proteins in the PfDCP2 interactome (Supplementary Data 4, Table S4i), including PfDOZI and constituents of the CCR4-NOT complex (NOT1, NOT1-G, and CAF40). The PfDCP2 interactome is enriched in GO terms related to the mRNA catabolic process. In comparison, the PfGBP2-GFP interactome from stressed schizonts included PfDOZI, PfCITH, eIF4A, PABP1, Alba proteins, translation initiation factors, and components of the 40 S ribosome, but they were devoid of proteins of the RNA decay pathway (Supplementary Data 4, Table S4k), suggesting the partition of PfGBP2 into SG-like mRNPs. Moreover, PfDCP2 and PfGBP2 did not precipitate each other, and their interactomes had a minimal overlap, only sharing the core element DOZI (Fig. S12).

These PfDOZI interactome studies indicate that PB-like and GCG-like RNP granules were more predominant in schizonts and gametocytes, respectively. Reciprocal pulldown analyses confirmed PfDOZI as a shared component in different mRNPs and supported the existence of PB- and SG-like sub-complexes in the PfDOZI interactomes. Stress conditions could lead to substantial remodeling of the PfDOZI complexes, suggesting the formation of SG-like mRNPs.

### *Pfdozi* disruption compromises the parasite’s stress responses

**Fig. 8:**
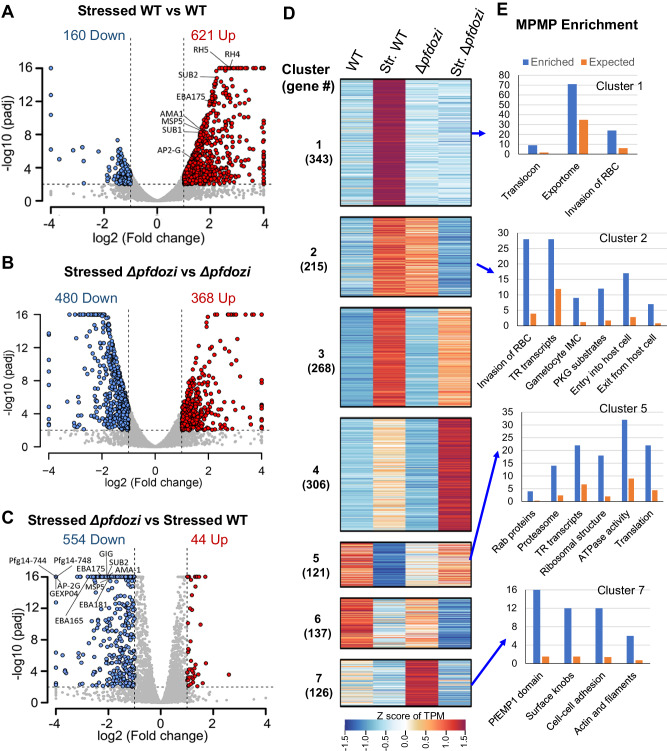
Transcriptomic analysis of the 3D7 and *Δpfdozi* schizonts under normal growth conditions and nutrient deprivation. **A**–**C** Volcano plots showing differential gene expression in schizonts at 40 hpi grown under nutrient stress vs. normal conditions in the WT 3D7 (A) and *Δpfdozi* parasites (**B**). A comparison in gene expression under nutrient stress conditions between *Δpfdozi* and WT 3D7 parasites is shown in **C**. For all the volcano plots, each dot represents one gene and is displayed as log_2_ (fold-change) on the *x*-axis and -log_10_(*Padj*) in the *y*-axis. All -log_10_ (*Padj*) above 16 were set as 16. Statistically upregulated and downregulated genes for each comparison are shown as red and blue dots, respectively. **D** Heat map depicting the *K*-mean clustering of differentially expressed transcripts in WT and *Δpfdozi* parasites grown under normal or stress conditions. The number of transcripts in each cluster was indicated in the parenthesis. Str., stressed. **E** Bar graphs showing the enriched pathways in Cluster 1, 2, 5, and 7 from MPMP analysis. The *y*-axis indicates the number of genes identified (blue) compared to the number expected (orange) for each enriched pathway.

With the evidence of PfDOZI’s participation in SG-like mRNPs in *P. falciparum*, we wanted to determine if *pfdozi* disruption affects the parasite’s stress response. We investigated the response of the schizonts to nutrient starvation as the stress condition. Although the WT and *Δpfdozi* schizonts had similar numbers of transcripts (781 and 848, respectively) with significantly altered levels under nutrient starvation, the proportions of genes with increased or decreased levels were markedly different (Fig. 8A, B). The stressed WT parasites showed almost four-fold more genes with increased abundance (621) than those with less abundant transcripts (160), indicating that more transcripts were protected from degradation during nutrient stress (stressed WT vs. control WT, Supplementary data 5: Table S5a). Under nutrient stress, *Δpfdozi* schizonts had an opposite trend in mRNA abundance: 368 and 480 genes with more and less abundant transcripts, respectively (stressed *Δpfdozi* vs. control *Δpfdozi*), suggesting compromised protection of transcripts in *Δpfdozi*. These results showed that the WT and *Δpfdozi* parasites differed drastically in steady-state mRNA levels in response to nutrient stress (*P* < 0.00001, *χ*^2^ test). Furthermore, a direct comparison of the transcriptomic changes between the WT and *Δpfdozi* under the stress conditions revealed that *pfdozi* disruption led to 598 genes with altered transcript levels, 92.6% of which showed reduced transcript levels (Fig. 8C, Supplementary Data 5: Table S5a), further highlighting the *Δpfdozi*’s defects in stabilizing transcripts.

To further illustrate the effect of nutrient stress on mRNA metabolism, mRNAs with altered levels were classified into seven clusters (Fig. 8D, Supplementary Data 5: Table S5b). Cluster 1 (*n* = 343) mRNAs were more abundant in stressed WT but no apparent change in the stressed *Δpfdozi*, while Cluster 2 (n = 215) mRNAs were more abundant in stressed WT but less in stressed *Δpfdozi*. MPMP analysis indicated that RBC invasion and protein export were significantly enriched in these two clusters (Fig. 8E, Supplementary Data 5: Table S5c). In addition, as retention of spent culture medium is routinely used to induce gametocytes, the increased levels of genes involved in sexual commitment (*ap2-g*) and earlier gametocyte development (*pfg14-744* and *pfg14-748*) in the WT parasites is consistent with the induction of gametocytogenesis (Fig. 8A, E). However, *ap2-g* and several early gametocyte genes were among the top genes with reduced transcript levels in stressed *Δpfdozi*, consistent with the lower sexual conversion rate observed in *Δpfdozi*. Cluster 5 includes 121 downregulated transcripts in stressed WT but with no significant change in stressed *Δpfdozi*, which are enriched in Rab proteins, proteasome machinery, ATPase activity, and translation-related (translationally repressed transcripts, ribosome, and translation), suggesting that these cellular activities (transport, protein degradation, and translation) were halted during stress. Collectively, these results indicated that the stress conditions applied caused the schizonts to shift to preserve transcripts involved in RBC invasion and gametocytogenesis, whereas this response was compromised in the *Δpfdozi* parasites.

## Discussion

This study demonstrated that PfDOZI carries out divergent, stage-specific functions in mRNA decay, repression, and storage. Previous work in *P. berghei* has identified that the PbDOZI RNPs in gametocytes resemble the germline-specific P granules, which stabilize the mRNAs required for ookinete development^26,27^. *Pbdozi* deletion does not affect the asexual IDC or gametocyte development but abrogates post-zygotic, ookinete development^23,26^. In this study, we identified similar GCG-like PfDOZI complex(es) in *P. falciparum* gametocytes. While the PfDOZI complex has retained a similar function in preserving mRNAs needed for ookinete development, it has attained additional functions to stabilize transcripts needed for late gametocyte development in *P. falciparum*, as reflected in the expanded PfDOZI regulon in gametocytes. Specifically, this includes genes required for cytoskeleton organization, motor activity, and gametocyte-specific crystalloid formation, which explains the defects in cytoskeleton rearrangements necessary for elongation during gametocyte maturation^33,42^. In addition, we identified that *pfdozi* disruption led to a similar number of genes with increased transcript abundance, indicating an equally significant role of PfDOZI complex(es) in RNA degradation, at least during the early stage of gametocytogenesis.

In stark contrast to the more restricted functions of DOZI and its associated components (e.g., CITH and Alba4) in sexual development in the rodent parasites^10,23,26^, our study uncovered a significant role of PfDOZI in mRNA metabolism during the IDC with the most noticeable effect observed in the ring and schizont stages. In the early IDC, the PfDOZI complex plays a major role in stabilizing transcripts involved in host cell modification, while its major function switches to RNA degradation in schizonts. The invasion-related genes, transcribed much earlier in trophozoites^34^, may have to be translationally repressed and degraded before being translated “just-in-time” in the late schizont stage when they are needed. In *P. yoelii*, deletion of *pyalba4*, a stable component of the DOZI complex, led to a similar alteration in gene expression in schizonts with 251 upregulated and only four downregulated transcripts^10^. These converging findings from the two parasites indicate a more prominent role of the DOZI complexes in RNA decay during the late IDC, which also explains the peak-level gene knockdown achieved in the schizont stage with the TetR-DOZI gene regulation system^43^. Collectively, we have demonstrated that PfDOZI, like its metazoan orthologs, is a versatile regulator of mRNA metabolism, and these functions are dynamically regulated during development with stage-specific characteristics.

The actions of PfDOZI on the protection or decay of diverse sets of target mRNAs are likely bestowed by partner proteins in the complexes. In yeast, Dhh1 participates in the organization of PBs through association with mRNA decay factors such as decapping and deadenylation enzymes^12^, whereas Dhh1 is also associated with SGs to serve as a depot to triage translationally stalled mRNAs under certain stress conditions^44^. In yeast and mammalian cells, PB and SG can occur in the same cells and are functionally linked^36,45^. In *P. falciparum* schizonts, while the PfDOZI interactome is enriched in PB components involved in mRNA decay, it also includes components that are typically found in SGs, suggesting the co-existence of PB-like and SG-like granules in schizonts. Together with *pfdozi* disruption results, the interactome study suggests a primary role of the PfDOZI complex in mRNA decay in schizonts. Reciprocal pulldown studies with PfDCP2 and PfGBP2 suggest the partition of PfDOZI into different sub-complexes, which may carry out distinct functions of RNA stabilization and degradation. Under stress conditions, the PfDOZI interactome in schizonts included more SG-related terms, consistent with the canonical definition of SGs. In gametocytes, the PfDOZI interactome is enriched in SG and GCG components, similar to the PbDOZI complex in *P. berghei* and Alba4 complex in *P. yoelii*^10,26^, suggesting a primary function of these GCG-like mRNPs to stabilize the translationally repressed mRNAs instead of translation repression since the deletion of either *pbdozi* or *pbcith* led to the destabilization but not the translation of these transcripts^26^. Furthermore, the PfDOZI interactome in gametocytes also contains classical PB components (e.g., CCR4-NOT complex), which is consistent with the RNA decay function of the PfDOZI complex in mid-stage gametocytes. Taken together, *P. falciparum* organizes RNPs similar to eukaryotic PBs, SGs, and GCGs, and their dynamics indicate a more prominent role of RNA decay in asexual stages and RNA preservation in sexual stages. While we provided limited evidence supporting that the distinct functions of PfDOZI were performed by different sub-complexes, future functional studies are needed to dissect these complexes.

The formation of PB-like granules to degrade transcripts in the asexual IDC and GCG-like granules to preserve transcripts during gametocyte development in malaria parasites is functionally parallel to PBs in the cytoplasm of metazoan somatic cells and GCG-like granules in germ cells or early embryos^12,13,21,35,46,47^. This analogy indicates that these early-branching protozoans have retained similar post-transcriptional regulatory mechanisms during evolution. Yet, there are also considerable variations and unique adaptations in the RNP granules in malaria parasites. In yeast, PB-mediated mRNA decay occurs by consecutive deadenylation, decapping, and ultimately 5’ → 3’ exonucleolytic degradation by the cytoplasmic 5’→3’ exoribonuclease XRN1^12^. Although we consistently detected in the PfDOZI interactomes the deadenylase complex, the decapping enzymes, and the decapping activator proteins (LSm1-7) with varying abundance depending on developmental stages and stress conditions, the XRN1 homolog (PF3D7_1106300) was not co-purified. This suggests that XRN1 might be loosely associated with the PfDOZI complex or the PB-like granules in *P. falciparum* carry out mRNA storage functions, as shown for the yeast and human PBs^48,49^. This assumption is consistent with the predominantly increased transcripts during nutrient starvation, even though PB components were remarkably enriched. Another distinction of the *Plasmodium* SG-like granules is the inclusion of the Alba family proteins^10,26^. *Plasmodium* parasites encode four Alba proteins^31,50^; all were reliably identified in the PyAlba4 interactome^10^, and all but Alba2 were present in the PfDOZI interactome under stress-free conditions. The participation of Alba proteins in cytoplasmic RNPs to regulate stability and translation of specific target mRNAs appeared to be an evolved function in the lineages of Chromalveolata and Excavata clades^10,51^. Interestingly, the PfDOZI complex regulates the stability and/or translation of egress and invasion-related genes in schizonts, a subgroup of which was the targets of PfAlba1^11^, suggesting Alba and other RBPs in the cytoplasmic RNPs are needed to recruit specific target mRNAs during development. Notably, although we identified potential stage-specific mRNA targets for PfDOZI complex through RNA-seq and RIP-seq analyses, the overlap of the two datasets was not extensive (Fig. 6). This disparity may reflect (1) that parts of the PfDOZI complex-associated mRNAs are not direct targets of PfDOZI but bound by other RBPs in the complex or in proximity of the complex and (2) that *pfdozi* deletion only affects the abundance of a fraction of the targets. Thus, the PfDOZI mRNA targets desire further refinement using more advanced method such as enhanced crosslinking and immunoprecipitation (eCLIP)-seq analysis.

While PBs are traditionally considered to form under normal conditions, and SGs arise under stresses, recent proteomic studies indicate that interactions among many SG components are constitutive in stress-free conditions^52^. The identification of SG components in schizonts under normal growth conditions may indicate constitutive SG assembly in a stress-free situation, whereas gametocytogenesis has always been linked to environmental stresses. To capture the dynamics in SG assembly under stress conditions, we subjected schizonts to nutritional deprivation and analyzed the changes in protein and mRNA compositions of the RNPs. Nutrient starvation is associated with translational inhibition and promotes SG and PB assembly. Indeed, we observed stress-induced enrichment of not only the SG components but also the PB components. This substantial remodeling of the RNPs during nutrition starvation conditions is consistent with a more prominent role of mRNA storage for the RNPs, as nutrient starvation resulted in nearly 600 upregulated transcripts. As expected, *pfdozi* disruption impaired the formation of these RNPs and diminished the protective effect on the target mRNAs (Fig. 7). Given the unifying role of PBs and SGs for mRNA storage under stress conditions, when mRNAs can exit and reenter translation^53,54^, it is likely that the malaria parasite uses similar mechanisms to store mRNAs during stress.

In mammalian cells, SG assembly induced by stress conditions is part of the conserved integrated stress response and involves phosphorylation of eIF2α^54,55^. Depending on the stress conditions (e.g., heat shock, nutrient deprivation, or drugs), PBs and SGs may form sequentially^36^, and the compositions of the assembled RNPs can vary considerably, reflecting the stress-specific needs of the cells^56^. Under starvation conditions, eIF2α was substantially enriched in the PfDOZI interactome (Supplementary Data 4: Table S4H), suggesting that the malaria parasite may use a similar pathway for the stress-induced organization of SGs. Also, the starvation-induced SGs were devoid of 40 S rps proteins and RACK1, which resembles the SG compositional changes in mammalian cells during chronic starvation^39^, confirming translational blockade of the stored mRNAs. Of relevance, the antimalarial drug artemisinin induces an ER stress response in the parasite with a hallmark of eIF2α phosphorylation, and resistance to these drugs is associated with elevated eIF2α phosphorylation, resulting in a temporary halt of development and latency^57,58^. During their life cycle, malaria parasites are exposed to different types of stresses (fever, starvation, drugs, etc.) and may use a shared crisis-response mechanism^59^. Therefore, the elucidation of PfDOZI-mediated responses to different stress conditions not only may unravel novel drug resistance mechanisms but also may define new targets for malaria control strategies.

**Fig. 9:**
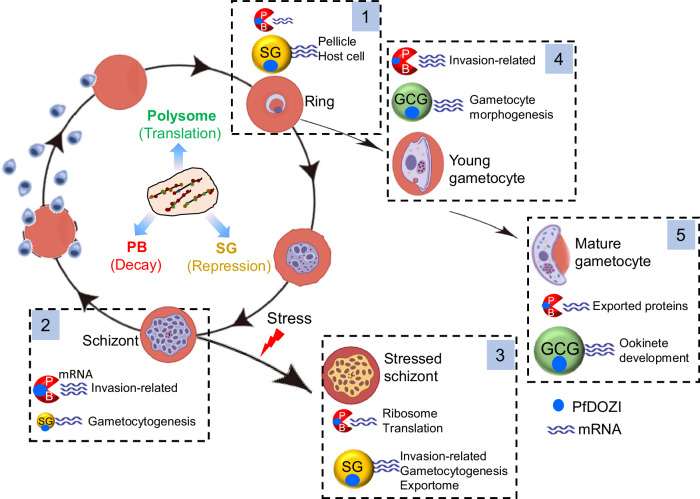
A model for the dual roles of PfDOZI in post-transcriptional regulation. The intraerythrocytic developmental cycle and gametocyte development are illustrated. During parasite development, cytoplasmic mRNAs are dynamically associated with different mRNPs and transit between polysome, PB, and SG to be translated, degraded, and stored, respectively. Boxes 1–5 show the partition of PfDOZI into PB-, SG-, and GCG-like mRNP granules in schizonts, stressed schizonts, and young and mature gametocytes, respectively. The sizes of mRNPs indicate the major or minor functions of the PfDOZI-associated mRNPs. The size of the PfDOZI (blue dot) indicates the relative proportion of PfDOZI participating in the respective mRNPs (not to scale). Major target mRNAs associated with the different mRNPs at different stages are highlighted. This figure was created with Biorender.

Our comprehensive omics studies during normal and stress conditions of the parasites allowed us to propose a model to illustrate the opposing actions of PfDOZI on mRNA metabolism in a stage- and transcript-specific manner in *P. falciparum* (Fig. 9). In the schizont stage, PfDOZI is predominantly localized in PB-like RNPs to degrade invasion-related genes and prevent their premature expression, while a minor proportion of PfDOZI participates in SG-like RNPs to preserve mRNAs needed for gametocytogenesis in cells committed to sexual development. Nutrient starvation induces stress responses and shifts the PfDOZI RNPs to a primary storage function to preserve translationally stalled mRNAs, whose translation can resume once the stress is lifted. Stress conditions also increase the cell proportion committed to sexual development, and PfDOZI is essential to protect these transcripts for gametocytogenesis. During early gametocyte development, PfDOZI complexes selectively degrade invasion-related mRNAs and preserve mRNAs for the morphogenesis of late-stage gametocytes. During gametocyte maturation, the PfDOZI complex, compositionally similar to metazoan GCGs, assumes a major role in the storage and translation repression of maternal mRNAs destined for ookinete development. Meanwhile, it adopts a minor role in mRNA decay to degrade mRNAs encoding exported proteins important for gametocyte sequestration so that mature gametocytes are released into blood circulation. The molecular mechanisms driving PfDOZI’s partition into different RNPs and its cooperation with the many core and accessory protein partners remain to be elucidated. The dependency of the malaria parasites on the DOZI complexes for development and stress responses opens new opportunities for therapeutically targeting these mRNA metabolic pathways.

## Methods

### Parasite culture, maintenance, and gametocyte induction

Parasites were cultured at 37 °C in a gas mixture of 5% CO_2_, 3% O_2_, and 92% N_2_ with type O^+^ RBCs at 5% hematocrit in RPMI 1640 medium supplemented with 25 mM NaHCO_3_, 25 mM HEPES, 50 mg/L hypoxanthine, 2 g/L glucose, 0.5% Albumax II, and 40 mg/mL gentamicin sulfate. For synchronization, the culture was initiated with purified schizonts using a Percoll step gradient, and the ring-stage parasites were treated with 5% D-sorbitol. A modified method of gametocyte induction was followed to obtain highly synchronous gametocyte cultures^60,61^. To generate stressed schizonts, ring-stage PfDOZI::GFP parasites were synchronized twice in two successive IDCs by 5% D-sorbitol treatment. The synchronized trophozoites were then used to set up culture at 2.0–2.5% parasitemia and 3% hematocrit. On the following day, when the parasitemia reached 10–12%, two-thirds of the medium was replaced by the fresh medium to generate stressed schizonts.

### Plasmid construction and generation of transgenic *P. falciparum* lines

To create a 3D7^DOZI::GFP^ line with the endogenous *pfdozi* gene tagged with a GFP tag at the C-terminus, a 581 bp 3’ fragment of the *pfdozi* gene (Pf3D7_0320800) encoding aa 297–433 of the PfDOZI protein was PCR amplified from the 3D7 genomic DNA using primers tagF and tagR (Supplementary Data 6: Table S6), and cloned into pBluescript SK to fuse with the *gfp* and pDT 3’UTR. The entire cassette containing the 3’ *pfdozi* fragment*, gfp*, and *pDT3*’ UTR was digested with *Bam*HI and *Not*I, and cloned into pHD22Y to generate the pHD22Y/DOZI::GFP plasmid. The same strategy was used to tag PfDCP2 and PfGBP2 (Fig. S11a). To disrupt the *pfdozi* gene, a 1009 bp 5’ fragment of the *Pfdozi* gene encoding aa 42–303 of the PfDOZI protein was amplified using primers Δ*pfdozi*-F and Δ*pfdozi*-R (Supplementary Data 6: Table S6) and cloned into pBluescript SK to fuse with the *gfp* and pDT 3’UTR. The 5’ *Pfdozi* cassette was then subcloned into the pHD22Y plasmid to produce the plasmid pHD22Y/DOZIΔCTD::GFP. To generate transgenic parasites, schizont-stage parasites were incubated with RBCs preloaded with the respective plasmids by electroporation^62^. Cultures were maintained under 2.5 nM WR99210 drug pressure until resistant parasites appeared. To enrich parasites with chromosomal integration of the transfected plasmids, parasites were subjected to two drug-on/off cycles. Parasites were then cloned by limiting dilution.

For genetic complementation and overexpression, the full-length *pfdozi* was amplified with cDNA using primers OVER-DOZIF and OVER-DOZIR (Supplementary data 6: Table S6) and cloned into tdTomato-pBluescript at *Bam*HI and *Avr*II. After digesting with *Spe*I and *Not*I, the released fragment was subcloned into pCC4-BSD to generate pCC4/dozi-tdT (Fig. S5a). Then, the plasmid was transfected into the Δ*pfdozi* K6 line and 3D7 for complementation and overexpression, respectively. At 48 h of transfection, parasites were cultured in the presence of 2.5 μg/mL BSD until parasites reappeared.

### Southern blot

Southern blot was performed to analyze the integration events in the transfected parasites. Briefly, parasite genomic DNA was isolated from the parasite pellet by proteinase K digestion and phenol/chloroform extraction. Genomic DNA was digested with *Hin*dIII, and separated on a 0.8% agarose gel. Then, the DNA was transferred to a nylon membrane, UV-crosslinked using a Stratalinker 2400 (Stratagene), and hybridized to a *pfdozi* probe. The probe was a 1151 bp *pfdozi* fragment amplified with primers DOZI probe F and DOZI probe R (Supplementary Data 6: Table S6) and labeled with DIG using the Probe synthesis kit (Roche). Hybridization and membrane washing were performed as described earlier^63^. Finally, the membrane was incubated with anti-DIG Fab fragments (Roche) at 1:10000 for 30 min. After adding the CSPD substrate (Roche), chemiluminescent signals were detected by exposing the membrane to an X-ray film.

### Western blots

Parasite lysates were prepared from synchronized parasites of different developmental stages. PfDOZI::GFP parasites were tightly synchronized by 5% D-sorbitol treatment and gametocytes were purified using a Percoll step gradient. Parasites were first released by lysing the erythrocyte membrane with 0.1% (v/v) saponin and washed twice with cold phosphate-buffered saline (PBS, pH 7.0). Protein concentration was measured using the Pierce BCA Protein Assay Kit (Thermo Fisher Scientific). Equal amounts of parasite lysates (40 μg) were separated by 10% SDS-PAGE and transferred to nitrocellulose membranes. PfDOZI::GFP expression was detected with a mouse anti-GFP antibody (1:1000, Invitrogen). PfAldolase was used as a loading control and detected by the rabbit anti-Aldolase antibodies (1:3000, Abcam). HRP-conjugated goat anti-rabbit/mouse IgG antibodies (Millipore) at 1:4000 were used as the secondary antibodies. The detected proteins were visualized using an enhanced chemiluminescence ECL detection kit (BioRad). To detect PfAMA1 and PfMSP5 expression, lysates of synchronized schizonts from WT and Δ*pfdozi* parasites were separated and probed with antibodies against PfAMA1 (1:1000) and PfMSP5 (1:1000) (obtained beiresources.org). To check the expression of GFP-tagged PfDCP2 and PfGBP2, western blots were conducted using parasite lysates from non-synchronized cultures with the anti-GFP antibody. Similarly, episomally expressed PfDOZI-tdTomato was tested by western blot using parasite lysates from non-synchronized cultures with the rabbit polyclonal anti-tdTomato antibody (1:3000, Novus Biologicals).

### Live fluorescence microscopy and indirect immunofluorescence assays (IFA)

The expression of PfDOZI::GFP and its subcellular localization were directly visualized using the GFP signal under the Olympus FV1000 confocal microscope using a 60× oil objective and processed by FV10-ASW imaging software. Nuclei were counter-stained with DAPI. For IFA, parasite cultures were coated on slides, allowed to air-dry, and then fixed by methanol: acetone (1:1) for 20 min at −20 °C. The samples were treated with 0.01% Triton X-100 for 5 min to permeabilize the parasite cell membrane and 5% BSA in PBS for 30 min at room temperature to block non-specific binding sites. Subsequently, the slides were incubated with primary antibodies in 3% BSA/PBS at 4 °C overnight and then secondary antibodies at room temperature for 1 h. Depending on the purpose, the primary antibodies included anti-PABP1 (1:1000)^32^, anti-Alba4 (1:1000), anti-α-tubulin II (1:2000) for male gametocytes, anti-GFP (1:500, Invitrogen), anti-β-tubulin (1:250, Sigma), anti-PfAMA1 (1:250, MR4), and anti-PfMSP5 (1:500, MR4). Secondary antibodies were Alexa Fluor 488 or 594 conjugated goat anti-mouse or donkey anti-rabbit IgG antibodies. The slides were mounted with Vectashield antifade mounting medium with DAPI and observed under an Olympus FV1000 confocal microscope. ImageJ software was used to measure the fluorescence density.

### Flow cytometry

To measure the parasitemia of 3D7 or *Δpfdozi* transgenic parasite lines, 5 μL of the culture were stained in 95 μL of Dulbecco’s PBS (DPBS, Gibco) supplemented with 2× SYBR Green for 20–30 min at room temperature. The cell pellets were washed twice and resuspended in DPBS. The SYBR Green fluorescence was measured in a Guava easyCyte Flow Cytometer (EMD Millipore). Uninfected RBCs were used as a negative control. For each sample, 25,000 events were counted to establish an accurate parasitemia. Data were analyzed by the FlowJo software.

### Phenotypic analysis

Each *Δpfdozi*, complementation, and overexpression line was compared to the WT 3D7 parasite in three biological replicates. To measure parasite proliferation, parasites were tightly synchronized with 5% D-sorbitol and the cultures were initiated at 0.1% parasitemia. Parasitemia was monitored daily by flow cytometry for three successive life cycles. To determine cell cycle progression, parasite cultures after two rounds of consecutive synchronization were initiated at 1% parasitemia. Progression of parasites through the IDC was monitored using Giemsa-stained smears every 3 h. The number of merozoites produced per schizont was determined in mature schizonts (segmenters). For easier visualization of the merozoites, nuclei were counter-stained with Hoechst 33342 (20 μM) for 5 min, and the smears were observed under a light and fluorescent microscope^64^. To compare the viability of merozoites between parasite lines, invasion rates were determined by using either purified merozoites or schizonts^65,66^. To purify merozoites, synchronous parasite cultures reaching the mature schizont stage were treated with E64 for 5–6 h to block schizont rupture and allow parasites to mature. Then, cultures were resuspended in RPMI-1640, and merozoites were released by filtration through a 1.2 μm membrane filter. Purified merozoites were incubated with RBCs at 37 °C for 18–20 h. Invasion rates were determined by scoring the frequency of ring-infected erythrocytes by Giemsa staining. In parallel, invasion assays were set up by directly incubating purified mature schizonts with RBCs to allow schizont rupture and re-invasion.

### Transmission electron microscopy (TEM)

Gametocytes 12 days after induction from the WT 3D7 and Δ*pfdozi* lines were purified by centrifugation on a 35% and 50% Percoll step gradient. The cells were washed with the RPMI-1640 medium and fixed by resuspension in 2.5% glutaraldehyde in 0.1 M cacodylate buffer (pH 7.2) containing 2 mM CaCl_2_. Cells were then immediately centrifuged at 1700 g for 10 min to collect the pellet. Fresh fixative was added to resuspend the pellet and incubated at room temperature for at least 1 h. Then, they were fixed in 2% osmium tetroxide for 1.5 h and *en bloc* stained in 2% aqueous uranyl acetate for 1 h. The cells were embedded in Eponite 12 (Ted Pella, CA). Ultrathin sections were prepared, contrasted with uranyl acetate and lead citrate, and examined with a JEOL JEM1200 EXII transmission electron microscope (Peabody, MA).

### RNA isolation and real-time PCR

Parasites of designated developmental stages were harvested after lysis of the RBC membrane with 0.1% (v/v) saponin in PBS. Total RNA was isolated from the purified parasites using Quick-RNA MiniPrep kit (Zymo Research). The RNA was treated with the ezDNase^TM^ Enzyme (Invitrogen) to remove contaminating genomic DNA. RNA pellets were dissolved in 50 µL RNase-free water, and the RNA integrity was checked using the Agilent Bioanalyzer. Real-time RT-PCR was performed using the Faststart Universal SYBR Green Master (Roche) with primers listed in Supplementary data 6. The relative expression levels of interested genes at different stages were determined using the 2^-ΔΔCt^ method with the s*eryl-tRNA synthetase* (*STS*) gene (*PF3D7_0717700*) as the internal reference.

### RNA-seq analysis

RNA-seq analysis was performed to compare global gene expression between highly synchronized WT and the Δ*pfdozi* parasites at 10, 20, 30, and 40 hpi during the IDC. Due to the drastically reduced gametocytemia in the *Δpfdozi* parasites after day 6, RNA-seq analysis was only performed on day 5 gametocytes when sufficient numbers of gametocytes could be purified from the *Δpfdozi* parasites. To determine the responses of the parasites to the nutrition stress, RNA-seq analysis was conducted only on the schizont stage (40 hpi), given the prominent role of PfDOZI in the schizont stage. All RNA-seq analyses were performed using three biological replicates.

RNA sequencing libraries were prepared using the KAPA stranded RNA-seq library preparation kit (Roche) with 500 ng RNA from each sample. RNA was fragmented by heating at 94 °C for 8 min, and cDNA was synthesized using reverse transcriptase with random primers. Afterward, 3’ dTMP adapters were ligated to the 3’ dAMP library fragments. The library was amplified for nine cycles. The quality of the barcoded libraries was validated using the Agilent Bioanalyzer. The DNA 1000 Reagents were used to quantify the library sizes and confirm the absence of primer dimers. Libraries were quantified using a KAPA Universal Library Quantification Kit (Roche), and library concentrations were adjusted for library size. Pooled libraries were sequenced on an Illumina HiSeq 2500 to produce 150 bp paired-end reads.

Illumina adapter sequence removal and quality trimming of reads were performed using Trimmomatic. Only reads that had a minimum length of 50 base pairs were retained. Reads were then mapped to the *P. falciparum* 3D7 strain reference genome with HISAT2^67^. Differentially expressed genes are determined by DESeq2^68^ with *Padj* < 0.01 and absolute log_2_ fold-change higher than 1. The transcriptional profiles are normalized by TPM for multi-time points or condition comparisons. GO enrichment for differentially expressed genes after *pfdozi* disruption was performed on PlasmoDB (https://plasmodb.org/). Pathways were assigned using the MPMP database^69^, and *p*-values for enrichment were calculated with Fisher’s statistic.

### Analysis of RNA decay

To compare the genome-wide RNA decay rates in 3D7 and *Δpfdozi* parasites, parasite lines were tightly synchronized by sorbitol treatment for two consecutive cell cycles. Pure schizonts were obtained using the Percoll gradient and incubated with fresh RBCs for 4 h. Rings with a 4-h window were obtained by sorbitol treatment. At the schizont stage (30 h), the cultures were treated with 20 μg/mL actinomycin D, and samples were collected at 0, 5, 15, 45, 60, 180, and 300 min. Total RNA was extracted from parasites, and RNA-seq libraries were prepared as described above. An average of more than 8 million reads per sample (each time point with three biological replicates) were used to align to the *P. falciparum* genome using HISAT2^67^. Read normalization was performed using transcript per million (TPM). RNA decay curves were fit to a first-order decay model as previously described^70^.

### Purification of the PfDOZI/PfDCP2/PfGBP2 interactomes and mass spectrometry

To identify the proteins associated with PfDOZI, PfDOZI::GFP parasites in the schizont stage (normal or stressed) or day 5 gametocytes were purified as described above. For the PfDCP2 and PfGBP2 interactomes, normal and stressed schizonts of respective GFP-tagged parasite lines were used for IP. Parasite pellet was lysed with five volumes of pre-cooled lysis buffer (300 mM NaCl, 20 mM HEPES, pH 7.9, 20% v/v glycerol, 2 mM MgCl_2_, 0.2 mM EDTA, 0.1% NP40, and 0.5 mM DTT) containing a protease inhibitor cocktail (Roche). Then, the parasites were mechanically lysed using a homogenizer with 80–100 strokes. The supernatant was incubated with 100 μL of GFP-Trap beads slurry (ChromoTek) with gentle rotation at 4 ^o^C for 2 h. Meanwhile, an aliquot of the parasite lysate was incubated with the agarose beads (ChromoTek) as the control. Subsequently, the beads were washed with washing buffer (300 mM NaCl, 20 mM HEPES, pH 7.9, 2 mM MgCl_2_, 0.2 mM EDTA, 0.1% NP40, 0.5 mM DTT) three times. Finally, the protein complex was eluted with a buffer (pH 2.8) containing a primary amine and concentrated by Amicon Ultra centrifugal filters (Millipore).

Protein extracts were separated by SDS-PAGE and Coomassie-stained for visualization. The gel section was minced and de-stained before being reduced with DTT, alkylated with iodoacetamide (IAA), and finally digested with Trypsin/Lys-C overnight at 37 ^o^C. Peptides were extracted using 50/50 acetonitrile (ACN)/H_2_O/0.1% formic acid and dried in a vacuum concentrator (Labconco). Peptides were resuspended in 98% H_2_O/2% ACN/0.1% formic acid for liquid chromatography-tandem mass spectrometry (LC-MS/MS). Experiments were performed in triplicate. Peptides were separated using a 50 cm C18 reversed-phase HPLC column (Thermo) on an Ultimate3000 UHPLC (Thermo) with a 120 min gradient (2–32% ACN with 0.1% formic acid) and analyzed on a hybrid quadrupole-Orbitrap mass spectrometer (Q Exactive Plus, Thermo Fisher Scientific) using data-dependent acquisition in which the top 10 most abundant ions were selected for MS/MS analysis. Raw data files were processed in MaxQuant and searched against the current Uniprot *P. falciparum* 3D7 protein sequence database. Proteins were identified using the filtering criteria of 1% protein and peptide false discovery rate (FDR). Unique proteins were identified after controlling the FDR at 1% using the Significance Analysis of INTeractome (SAINT) algorithm^71^.

### RNA immunoprecipitation and sequencing (RIP-seq) analysis

The 3D7^PfDOZI::GFP^ parasite line was grown to the schizont stage. The parasites were isolated as described above, and the pellet was resuspended in a lysis buffer (150 mM KCl, 20 mM Tris-HCl, pH 7.7, 3 mM MgCl_2_, 0.1% Tween 20, 0.5 mM DTT) containing protease inhibitor cocktail (Roche) and RNase inhibitor (Invitrogen) for 1 h at 4 °C with rotation. Lysates were cleared by centrifugation and incubated with 50 µL slurry GFP-Trap agarose beads (ChromoTek) for 2 h at 4 °C. Then, 100 µL of supernatant was mixed with 1 mL of Trizol as the input sample. Subsequently, the beads were washed with ice-cold wash buffer to remove nonspecifically bound materials. RNA-coated beads were mixed with 1 mL of Trizol as the IP sample. Both the input and IP samples were mixed with 200 µL chloroform and clarified by centrifugation at 13,000 **×** *g* for 15 min at 4 °C. The upper aqueous phase was mixed with isopropyl alcohol and glycogen to precipitate the RNAs by centrifugation. Finally, the RNA pellet was washed with 70% ethanol and dissolved in nuclease-free water. The sequencing step was processed as described above. Enrichment of transcripts was determined by a Gaussian mixture model with a set of stringent criteria, including a read-depth coverage greater than 10, a fold enrichment ratio greater than 2, and posterior probabilities of higher than 0.95 in both replicates.

### Statistical analysis

The phenotypical comparison between 3D7 and *Δpfdozi* parasite lines was carried out using the GraphPad Prism 8.0 software. One-way or two-way ANOVA followed by Dunnett’s multiple comparisons test was used for statistical comparison between transgenic parasite clones with WT in all the phenotypical assays. A two-tailed unpaired *t*-test was performed to analyze the IFA data. The differential expression of RNA-Seq data was determined by DESeq2 analysis. The Bonferroni-adjusted *p*-value is utilized for conducting the GO term enrichment analysis of the differentially expressed genes. All the data were represented as mean ± standard deviation (SD). Unless indicated otherwise, *p* < 0.05 was taken as statistically significant.

### Reporting summary

Further information on research design is available in the Nature Portfolio Reporting Summary linked to this article.

### Supplementary information

Supplementary Information

Description of additional supplementary files

Supplementary Data 1

Supplementary Data 2

Supplementary Data 3

Supplementary Data 4

Supplementary Data 5

Supplementary Data 6

Reporting Summary

### Source data

Source Data

## Data Availability

The data supporting the findings of this study are available in the article and the Supplementary Information. Source data are provided in this paper. All mass spectrometry raw, RNA-seq, and RIP-seq raw data were made publicly available on common data repositories. The protein mass spectrometry data generated in this study have been deposited to the ProteomeXchange Consortium via the PRIDE partner repository with the dataset identifier PXD020862 and PXD051782. Transcriptomics data are accessible at the GEO repository via accession code # GSE189034. Source data are provided in this paper.
