# Supplementary material for: The DEAD-box RNA helicase PfDOZI imposes opposing actions on RNA metabolism in Plasmodium falciparum
Source: Nat Commun. 2024 May 3;15:3747. doi: 10.1038/s41467-024-48140-4 (PMC11068891; doi:10.1038/s41467-024-48140-4)
Supplement: Supplementary file 1 — Supplementary Information [file 41467_2024_48140_MOESM1_ESM.pdf]

The DEAD-box RNA helicase PfDOZI imposes opposing actions on RNA metabolism in *Plasmodium falciparum*

Min et al.

Supplementary Figures

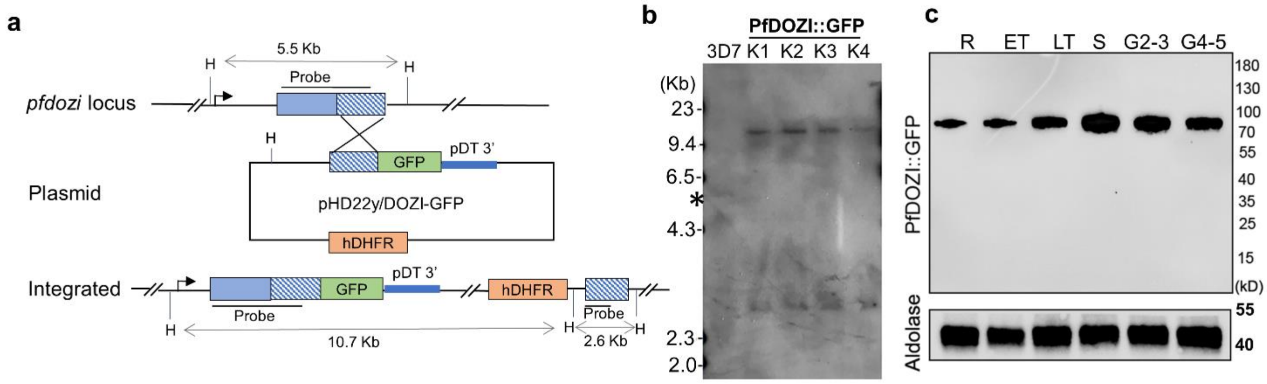

Figure S1. Tagging of *pfdozi* with the green fluorescent protein (GFP). a Schemes of the GFP-tagging of *pfdozi* at the C-terminus. Illustrated are the endogenous *pfdozi* locus on chromosome 3, the pHD22Y/DOZI-GFP plasmid, and the resultant integrated *pfdozi* locus with the integration of GFP from a single crossover event. The hatched boxes represent the region used for homologous recombination. The human dihydrofolate reductase (hDHFR) is the selectable marker. The fragment used as the probe for Southern blot analysis in **b** is indicated. H, HindIII. **b** Confirmation of pHD22Y-DOZI-GFP integration by Southern blot in four clones (K1-4). The detected bands are consistent with the expected size of 5.5 kb fragment for 3D7 (marked by an asterisk) as compared to the 10.7 and 2.6 kb bands for those with GFP-tagging. **c** PfDOZI-GFP fusion protein was detected an expected band around 75 kDa by Western blot using the anti-GFP antibodies. The bands show expression levels of PfDOZI in the ring (R), early trophozoite (ET), late trophozoite (LT), schizont (S), gametocytes at stage II-III (G2-3) and stage IV-V (G4-5), respectively. Aldolase serves as a loading control. Source data are provided as a Source Data file.

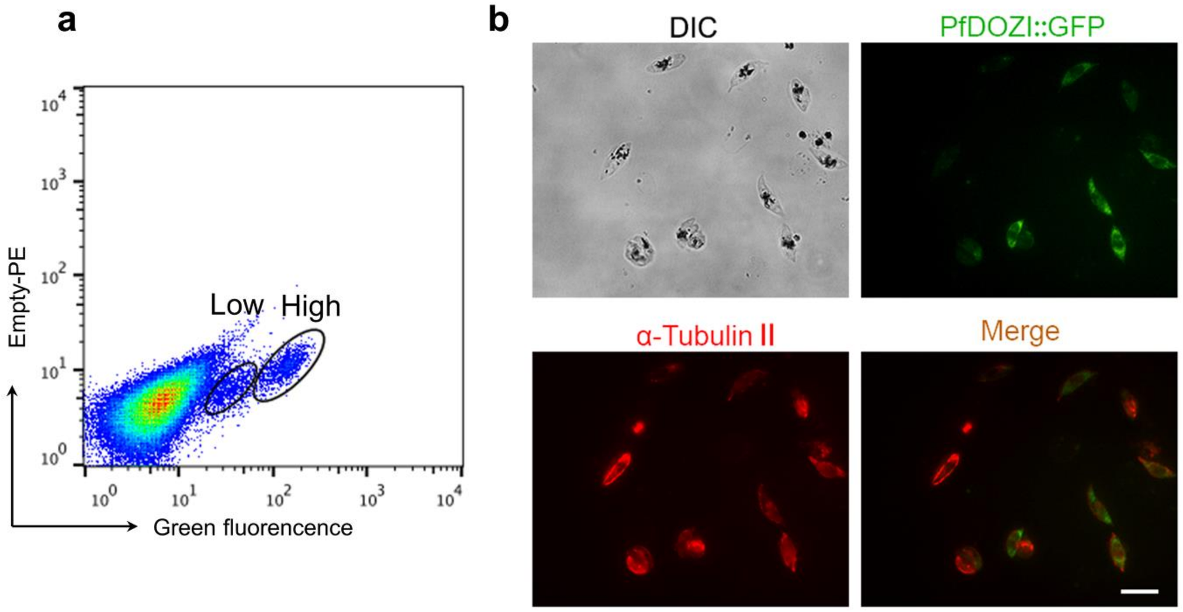

Figure S2. Preferential expression of PfDOZI::GFP in *P. falciparum* female gametocytes. **a** Flow cytometry analysis of gametocytes based on GFP fluorescence intensity. Two populations of gametocytes (circled) with low and high GFP fluorescence intensities were observed. The gating strategy was shown in Fig. S13. **b** PfDOZI::GFP gametocytes were purified and detected using an immunofluorescence assay with antibodies against α-tubulin II as the male gametocyte marker. DIC, differential interference contrast microscopy. Scale bar = 10 μm. Note that antibodies against α-tubulin II also stained the female gametocytes, but the fluorescent intensity is much weaker than in males. Similar results were obtained from three biological repeats.

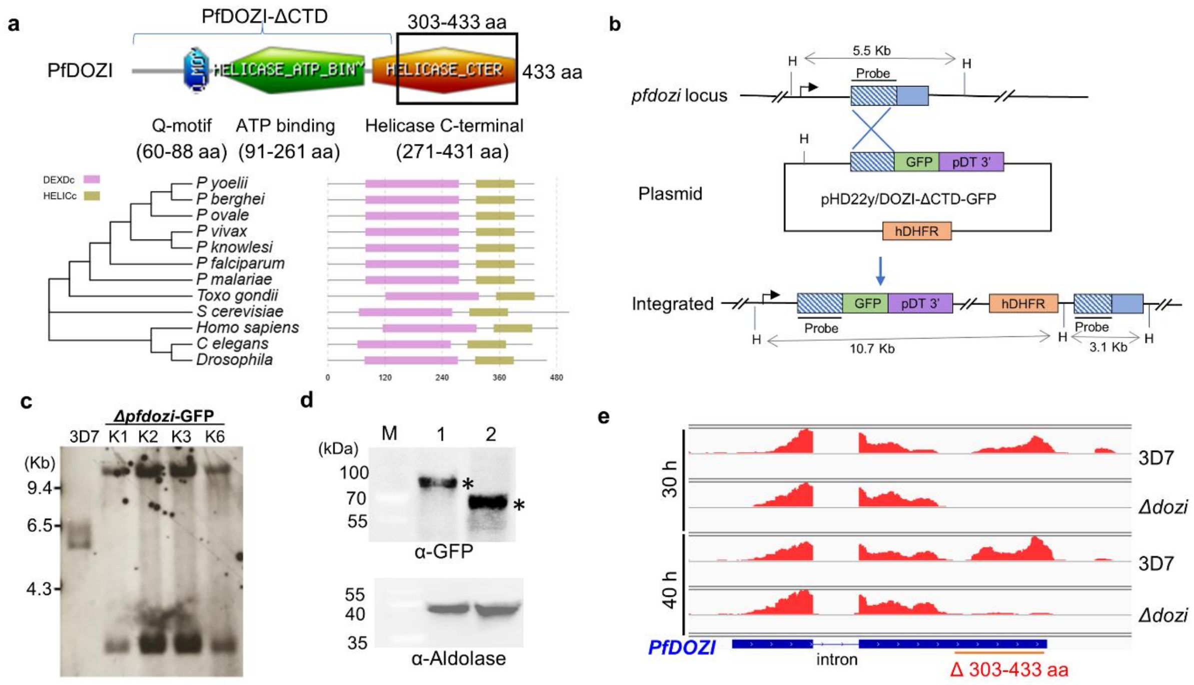

Figure S3. Disruption of the *pfdozi* gene. a PfDOZI domain analysis using Scan Prosite (upper panel). The span of each domain is shown in parenthesis. The black square indicates the C-terminal fragment (303-433 aa) deleted in *Δpfdozi*. Lower panel shows a neighbor-joining tree of DDX6 orthologs in different *Plasmodium* species and several model organisms. The analysis was performed using MEGA 7.0 and Evolview v3 software. Bootstrap values (500 replicates) were applied. Locations of the DEXDc (purple) and HELICc (yellow) domains are illustrated. **b** Schematic of the *pfdozi* disruption leading to the deletion of 130 amino acids from the C-terminus (PfDOZI-ΔCTD) using single-crossover homologous recombination in 3D7. A GFP tag was added to the truncated *pfdozi*. The fragment used as the probe for Southern blot analysis in panel C is indicated. **c** Southern blot analysis of four clones (K1, K2, K3, and K6). The band patterns are consistent with the expected size of 5.5 kb in 3D7 versus 10.7 and 3.1 kb in the disruptant lines. Clones K2 and K6 were selected for further studies. **d** Western blot with lysates of mixed parasite stages from PfDOZI::GFP and PfDOZI-ΔCTD::GFP parasites using the anti-GFP antibody. Asterisks (*) mark the PfDOZI-GFP (lane 1, ~75 kDa) and the PfDOZI-ΔCTD-GFP protein (lane 2, ~60 kDa). Anti-aldolase was used for loading control. M, molecular marker in kDa. **e** Integrative Genomics Viewer (IGV) snapshot depicting *PfDOZI* genome organization and comparison of RNA-seq results between wild-type 3D7 and *Δpfdozi* at 30 h and 40 h. Note the lack of reads mapping to C-terminal deletion. Background level reads were mapped to the disrupted region at 40 h, which might be due to promoter activity of the drug cassette as it is in the same orientation as the disrupted C-terminal fragment. Source data are provided as a Source Data file.

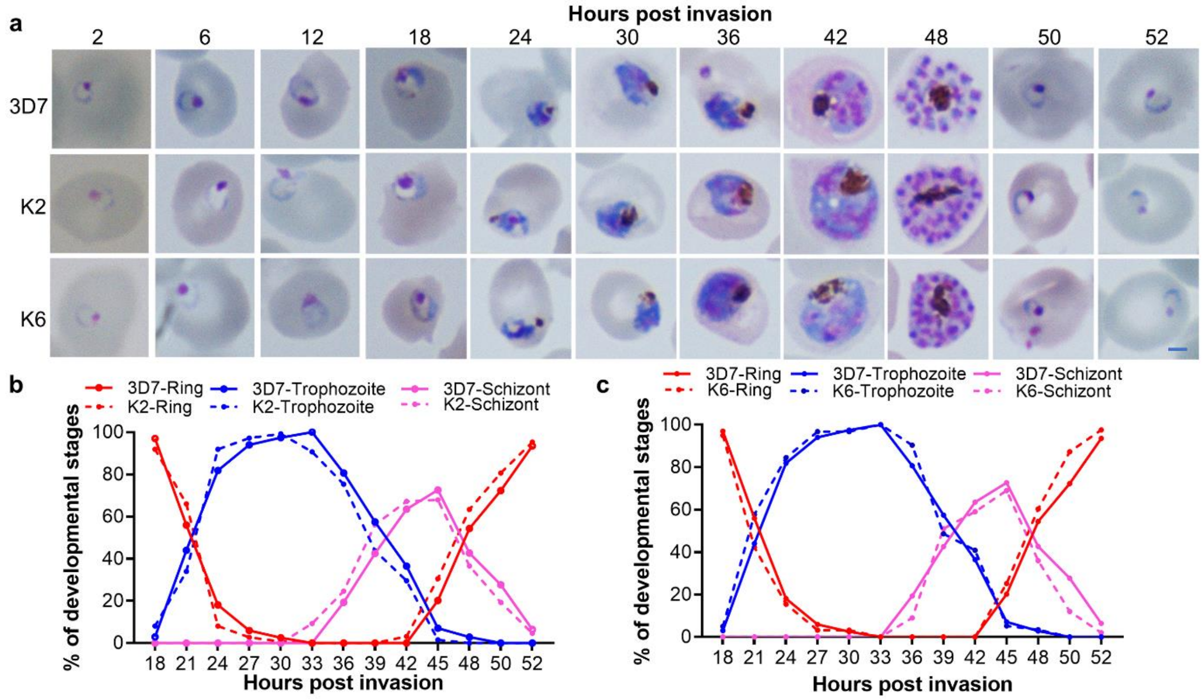

Figure S4. Disruption of *pfdozi* does not impact the intraerythrocytic developmental cycle (IDC) of the parasites. a Representative images of infected RBCs from Giemsa-stained thin smears observed by light microscopy at a 6-h interval. The wild-type 3D7 was compared to two *pfdozi* disruptant lines K2 and K6. Scale bar = 2 µm**.** Line charts comparing the proportions of the ring, trophozoite, and schizont stages through the IDC between the 3D7 (solid lines) and *Δpfdozi* parasite clones (dashed lines) K2 (**b**) and K6 (**c**). Blood smears were made every 3 h during the IDC to enumerate the different developmental stages. Similar results were obtained from two biological repeats. Source data are provided as a Source Data file.

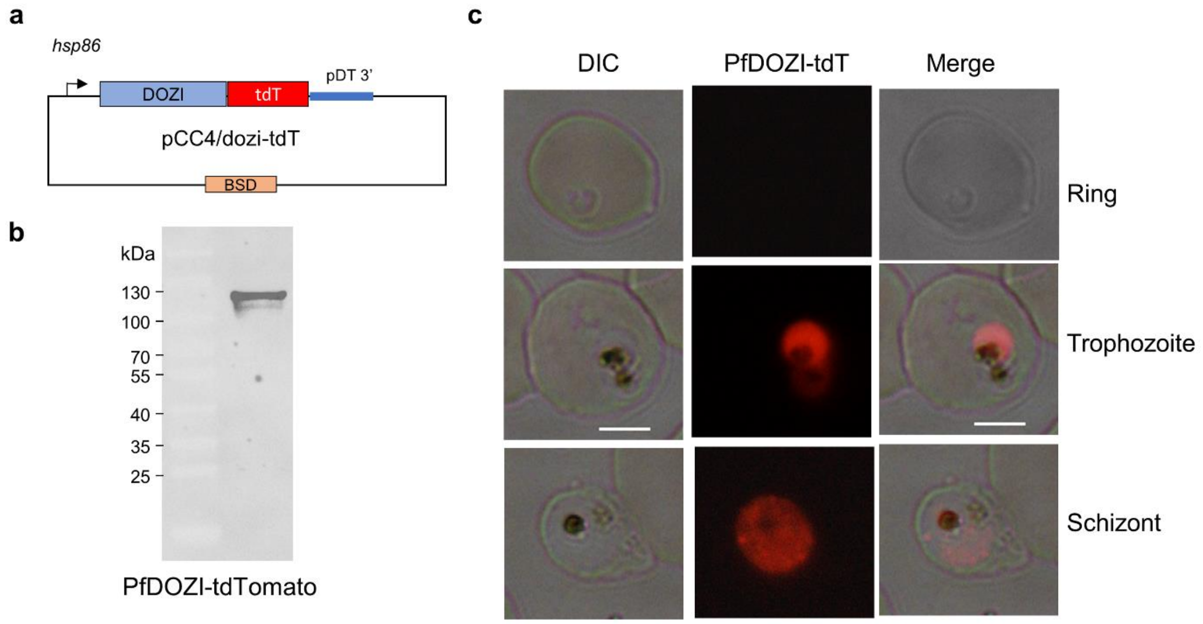

Figure S5. Complementation of *Δpfdozi* by episomal expression. a Plasmid construct used for *pfdozi* complementation and overexpression with the *pfdozi* C-terminus tagged with tdTomato (tdT). The PfDOZI-tdT was episomally expressed in the PfDOZI-ΔCTD parasite clone K6 (for complementation) or wild-type 3D7 (for overexpression). All parasites were selected under 2.5 μg/ml BSD. **b** Western blot analysis detected PfDOZI-tdTomato expression in the complementation line with parasite lysate of mixed asexual stages using the anti-tdTomato antibody. **c** Representative live images showing the PfDOZI-tdTomato expression in asexual erythrocytic stages. Scale bar = 5 μm. Similar results were obtained from two biological repeats.

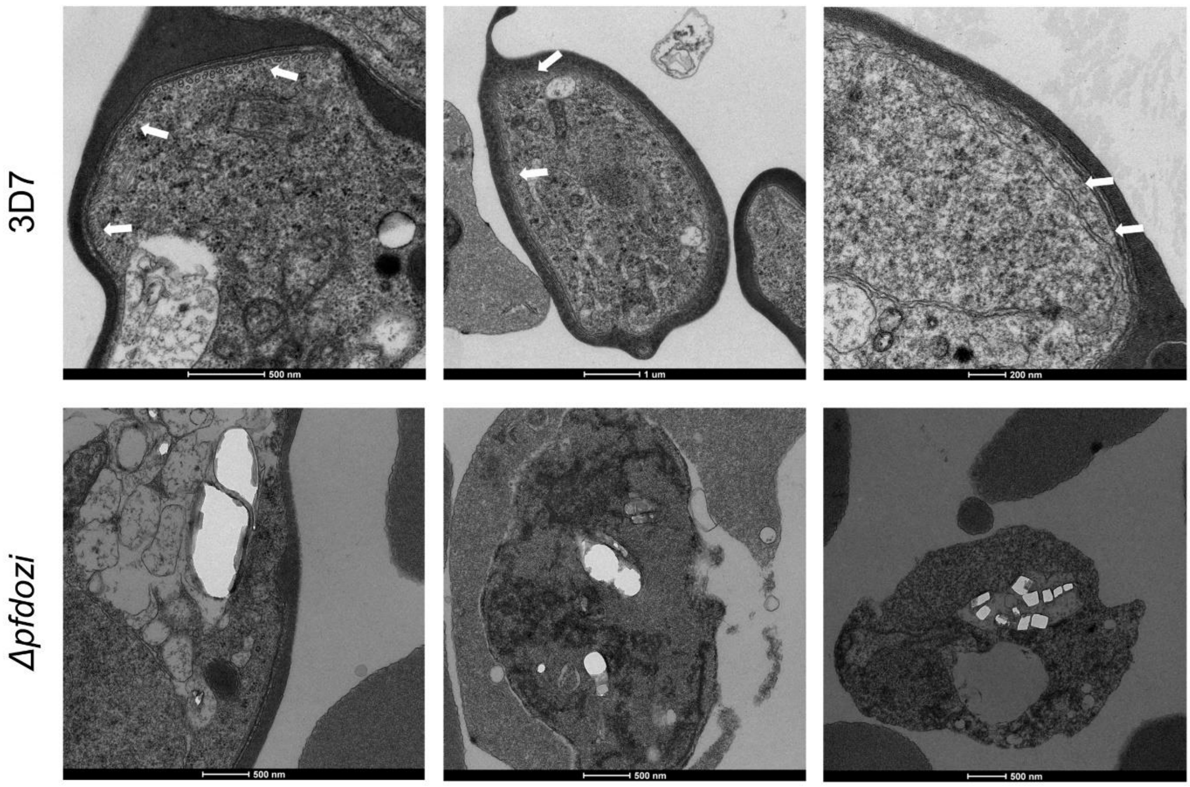

Figure S6. Additional transmission electron microscopy images of the wild-type 3D7 gametocytes (upper panels) and the *Δpfdozi* gametocytes. White arrows show the presence of subpellicular microtubules in the WT gametocytes. Similar results were obtained from two biological repeats.

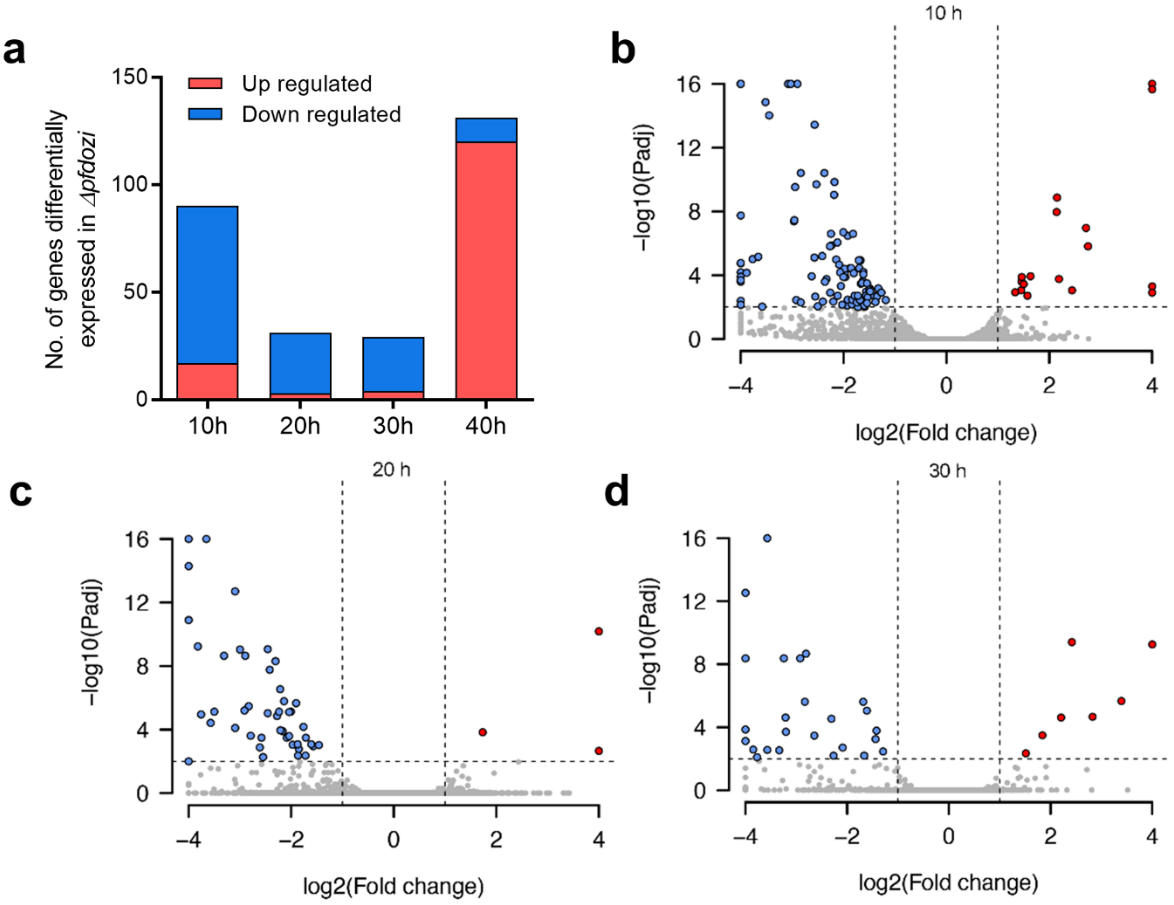

Figure S7. Transcriptomic changes in *Δpfdozi* during the IDC. a Bar graph showing the numbers of upregulated (red) and downregulated (blue) genes at 10, 20, 30, and 40 hpi. Note that multigene families (*var*, *rifin, stevor*, and *MC-2TM* were removed from analysis). **b-d** Volcano plots showing significantly (*Padj* < 0.01) upregulated (red) and downregulated (blue) transcripts in the *Δpfdozi* line compared to the corresponding 3D7 parasite at 10 **(b)**, 20 **(c)**, and 30 hpi **(d)**. –log10 (*Padj*) values above 16 were set as 16. The differential expression were calculated by DESeq2 analysis.

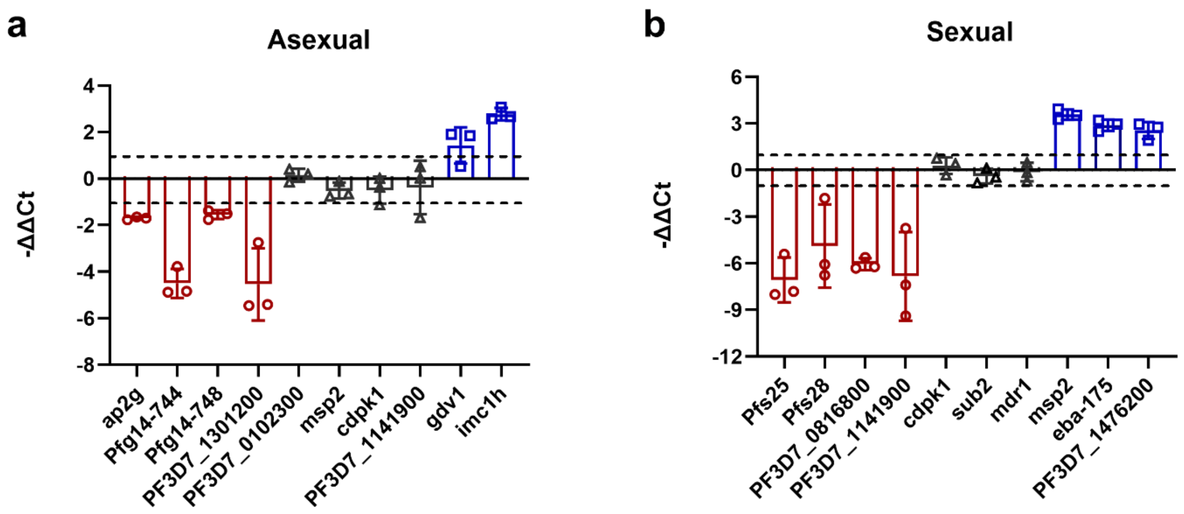

Figure S8. Verification of RNA-seq data by qRT-PCR. The bar graphs show qRT-PCR analysis of the indicated genes using total RNA isolated from 3D7 or *Δpfdozi* line in **(a)** asexual (40 h) and **(b)** sexual stages (day 5 after induction). Red, grey, and blue bars indicate genes that were less abundance, unchanged, and more abunandt in the *Δpfdozi* line, respectively, according to RNA-seq. All the values are represented as the relative expression in comparison to the PF3D7_0717700 gene. Error bars indicate the mean ± SD of three independent experiments. The relative expression level of each gene was calculated using the $2^{-\Delta\Delta Ct}$ method.

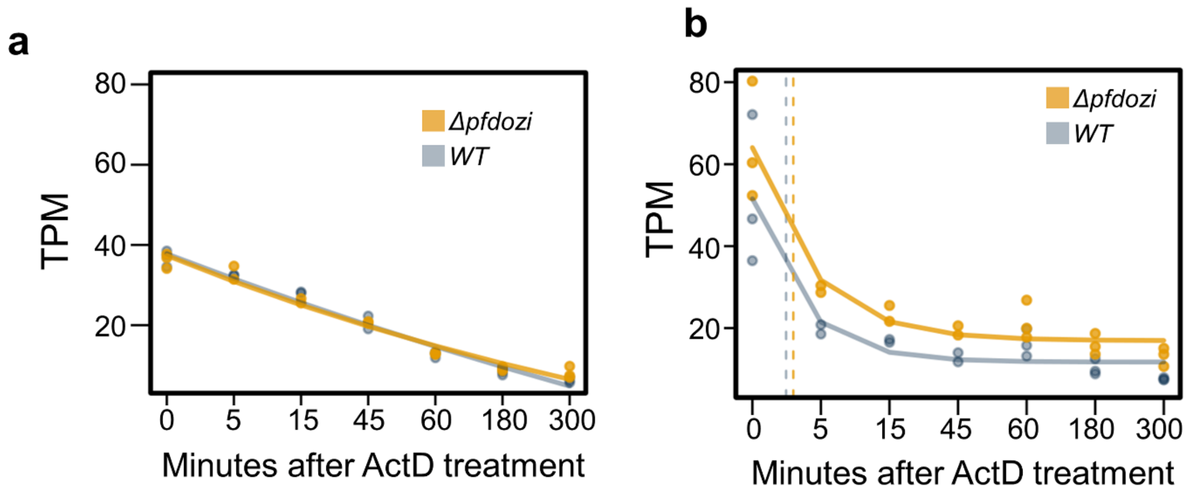

Figure S9. RNA decay rates in *Δpfdozi* and WT lines at the schizont stage. Synchronized schizonts at 40 h were treated with actinomycin D (ActD) and RNA was isolated at 5 – 300 min after treatment. RNA-seq analysis was performed. Normalized reads of transcripts (transcripts per million – TPM) were plotted against the time. Each dot on the graph represents the TPM value of one of three biological replicates. **a** Genome-wide transcript abundance. **b** 120 genes with increased transcript abundance in *Δpfdozi* from RNA-seq analysis.

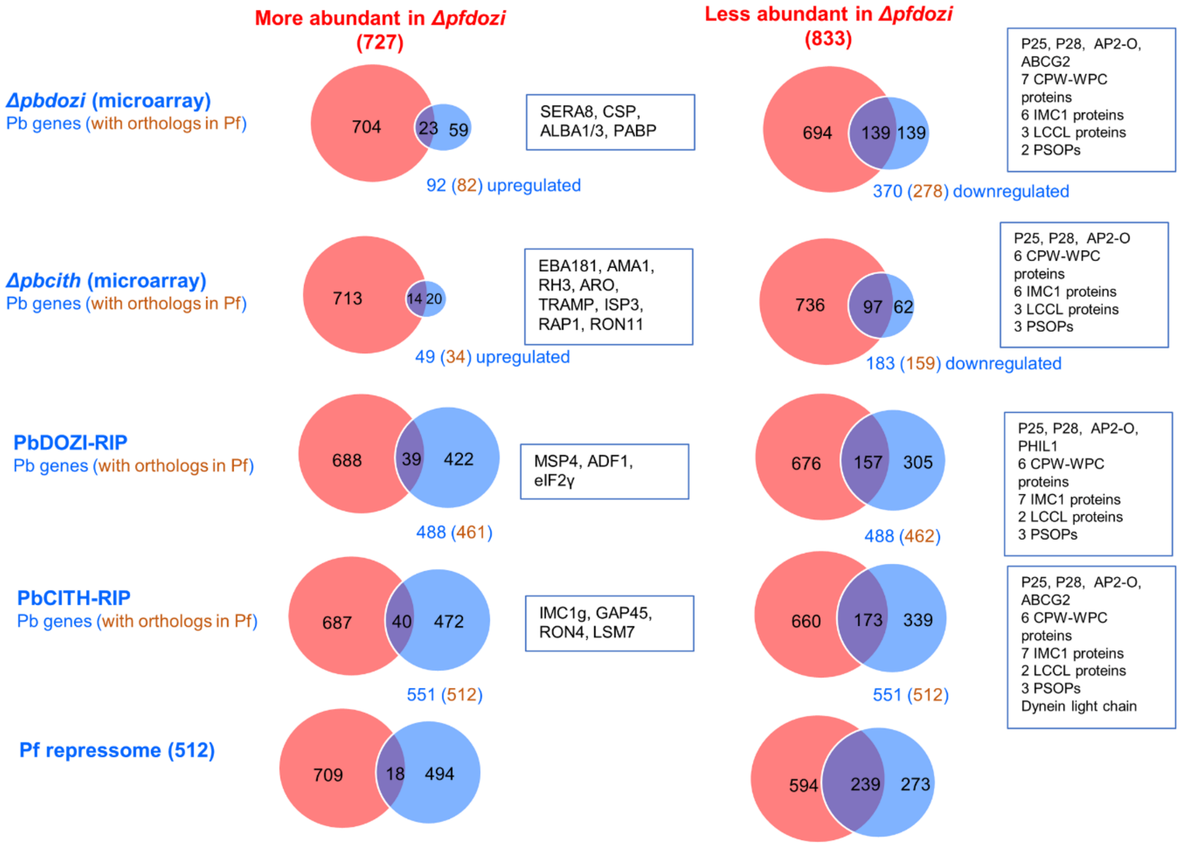

Figure S10. Venn diagrams showing the overlap of mRNAs upregulated or downregulated in *Δpfdozi* gametocytes with corresponding data from *Δpbdozi*, *Δpbcith*, PbDOZI-RIP, PbCITH-RIP, and the *P. falciparum* repressome. Shared genes and gene families are shown in the boxes. The *Δpbdozi* and *Δpbcith* microarray data were from Mair *et al.* [1-2]. The RIP data were from Guerreiro *et al.*[3]. The *P. falciparum* repressome data were from Lasonder *et al.*[4]. Please note that the more and less abundant gene numbers (727 and 833) excluded *var* genes.

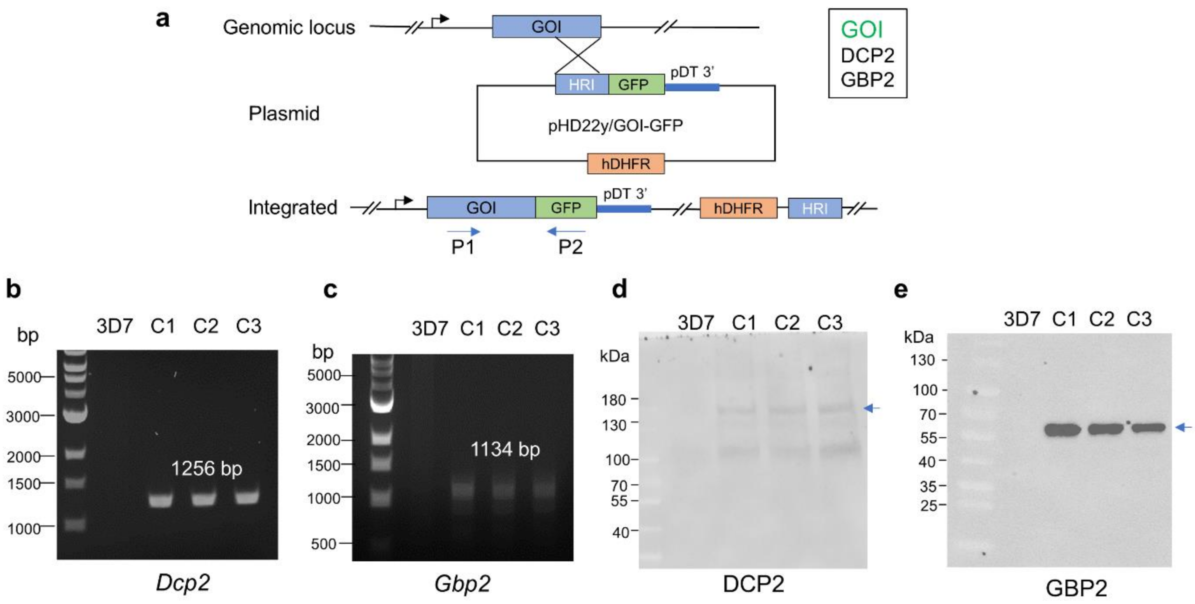

Figure S11. Tagging of DCP2 and GBP2 with the green fluorescent protein (GFP). a Schemes of the GFP-tagging of the endogenous gene locus at the C-terminus. The HRI represents the region used for homologous recombination. The human dihydrofolate reductase (hDHFR) is the selectable marker. GOI – gene of interest. P1 (Dcp1genoF or Gbp2genoF), and P2 (GFP-R) are the primers used for integration-specific PCR. **b-c** Confirmation of plasmid integration by PCR of genomic DNA from 3D7 (control) and three clones of gene tagging (C1, C2, and C3) using primer pairs P1 x P2. Correct integration of the plasmid results in the PCR products of 1256 bp and 1134 bp for *dcp2* (**b**) and *gbp2* (**c**), respectively. **d-e** GOI::GFP fusion proteins (marked by arrows) were detected by Western blot using anti-GFP antibodies. The expected fusion protein size is 165 and 57 kDa for DCP2-GFP and GBP2-GFP, respectively. Note a protein band of ~100 kDa was also detected in the DCP2-GFP tagged clones, which might be a processed protein product.

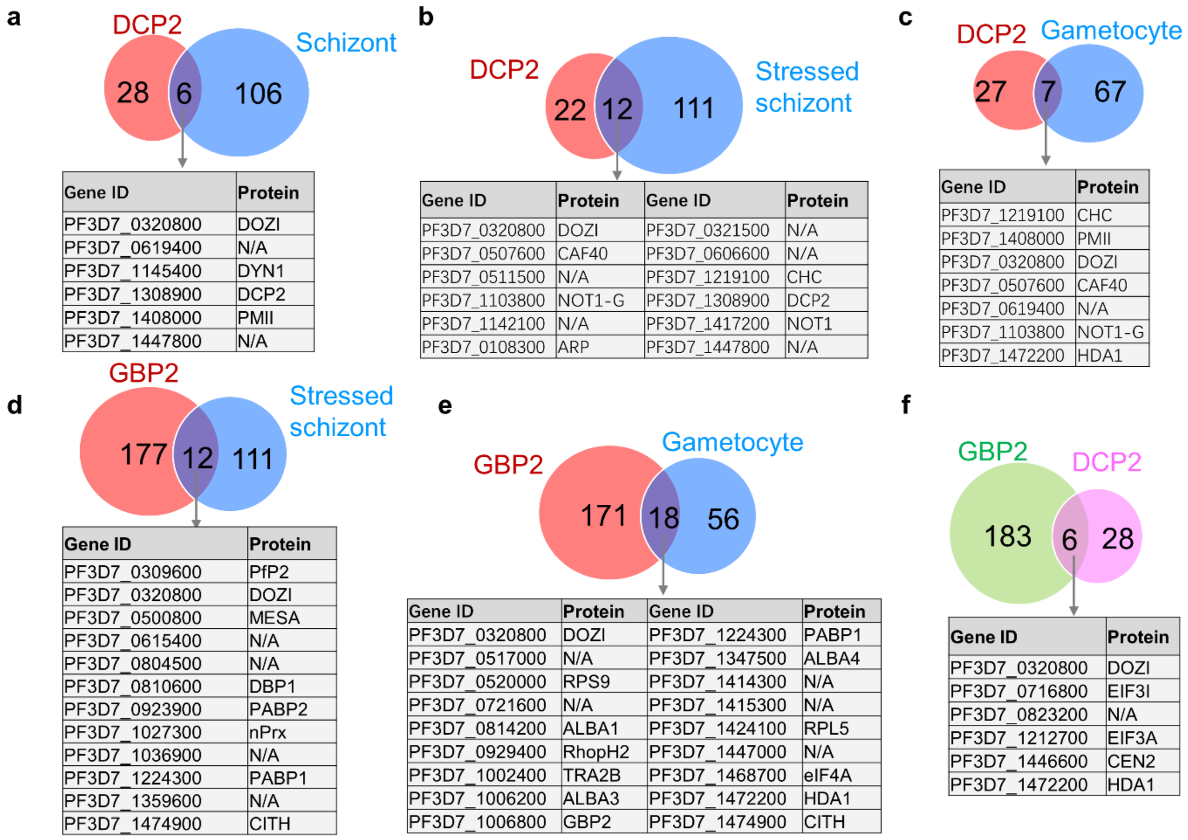

a

Gene ID	Protein
PF3D7_0320800	DOZI
PF3D7_0619400	N/A
PF3D7_1145400	DYN1
PF3D7_1308900	DCP2
PF3D7_1408000	PMII
PF3D7_1447800	N/A

b

Gene ID	Protein	Gene ID	Protein
PF3D7_0320800	DOZI	PF3D7_0321500	N/A
PF3D7_0507600	CAF40	PF3D7_0606600	N/A
PF3D7_0511500	N/A	PF3D7_1219100	CHC
PF3D7_1103800	NOT1-G	PF3D7_1308900	DCP2
PF3D7_1142100	N/A	PF3D7_1417200	NOT1
PF3D7_0108300	ARP	PF3D7_1447800	N/A

c

Gene ID	Protein
PF3D7_1219100	CHC
PF3D7_1408000	PMII
PF3D7_0320800	DOZI
PF3D7_0507600	CAF40
PF3D7_0619400	N/A
PF3D7_1103800	NOT1-G
PF3D7_1472200	HDA1

d

Gene ID	Protein
PF3D7_0309600	PfP2
PF3D7_0320800	DOZI
PF3D7_0500800	MESA
PF3D7_0615400	N/A
PF3D7_0804500	N/A
PF3D7_0810600	DBP1
PF3D7_0923900	PABP2
PF3D7_1027300	nPrx
PF3D7_1036900	N/A
PF3D7_1224300	PABP1
PF3D7_1359600	N/A
PF3D7_1474900	CITH

e

Gene ID	Protein	Gene ID	Protein
PF3D7_0320800	DOZI	PF3D7_1224300	PABP1
PF3D7_0517000	N/A	PF3D7_1347500	ALBA4
PF3D7_0520000	RPS9	PF3D7_1414300	N/A
PF3D7_0721600	N/A	PF3D7_1415300	N/A
PF3D7_0814200	ALBA1	PF3D7_1424100	RPL5
PF3D7_0929400	RhopH2	PF3D7_1447000	N/A
PF3D7_1002400	TRA2B	PF3D7_1468700	eIF4A
PF3D7_1006200	ALBA3	PF3D7_1472200	HDA1
PF3D7_1006800	GBP2	PF3D7_1474900	CITH

f

Gene ID	Protein
PF3D7_0320800	DOZI
PF3D7_0716800	EIF3I
PF3D7_0823200	N/A
PF3D7_1212700	EIF3A
PF3D7_1446600	CEN2
PF3D7_1472200	HDA1

Figure S12. Venn diagrams and tables showing the overlap of protein complexes. Protein pulldown and mass spectrometry analyses were performed with GFP-tagged DCP2, GBP2 and DOZI (blue). **a** DCP2 and PfDOZI schizont. **b** DCP2 and PfDOZI stressed schizont. **c** DCP2 and PfDOZI gametocyte. **d** GBP2 and PfDOZI stressed schizont. **e** GBP2 and PfDOZI gametocyte. **f** GBP2 and DCP2. Tables display the lists of shared proteins.

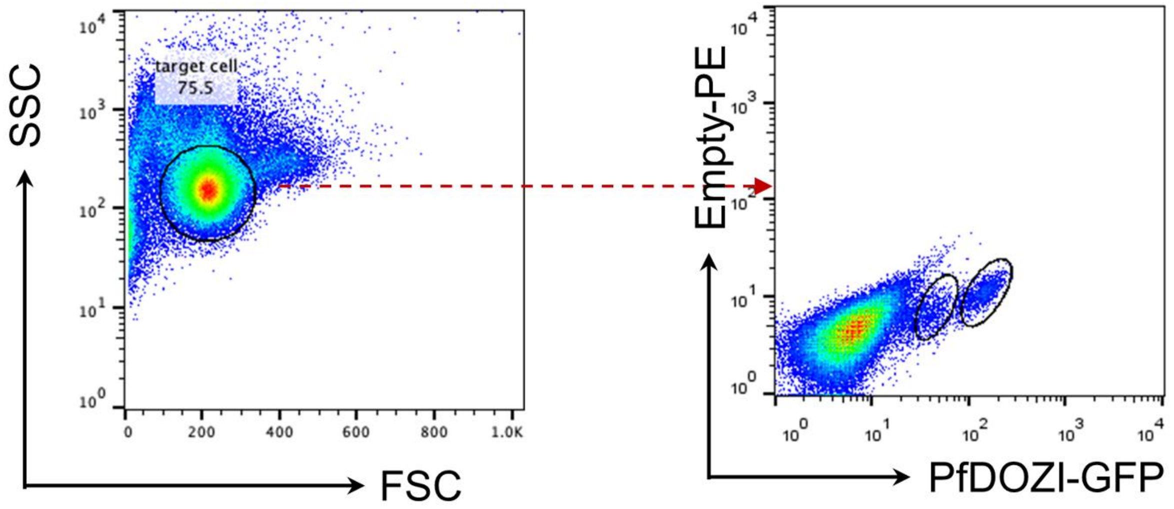

Figure S13. Gating strategies used for Fig. S2a. FCS and SSC were used to gate out targeted cells and were analyzed for GFP (PfDOZI) expression. PE channel was empty for the blank control.

Supplementary References

1 Mair, G. R. et al. Regulation of sexual development of Plasmodium by translational repression. *Science* **313**, 667-669, 1125129 (2006).

2 Mair, G. R. et al. Universal features of post-transcriptional gene regulation are critical for Plasmodium zygote development. *PLoS Pathog* **6**, e1000767, 1000767 (2010).

3 Guerreiro, A. et al. Genome-wide RIP-Chip analysis of translational repressor-bound mRNAs in the Plasmodium gametocyte. *Genome biology* **15**, 49 (2014).

4 Lasonder, E. et al. Integrated transcriptomic and proteomic analyses of P. falciparum gametocytes: molecular insight into sex-specific processes and translational repression. *Nucleic Acids Res* **44**, 6087-6101 (2016).
