# Supplementary material for: The DEAD-box RNA helicase PfDOZI imposes opposing actions on RNA metabolism in Plasmodium falciparum
Source: Nat Commun. 2024 May 3;15:3747. doi: 10.1038/s41467-024-48140-4 (PMC11068891; doi:10.1038/s41467-024-48140-4)
Supplement: Supplementary file 2 — Description of additional supplementary files [file 41467_2024_48140_MOESM2_ESM.docx]

**Description of additional supplementary files**

**Supplementary data 1**

**Title:** RNA-seq analysis of 3D7 and *Δpfdozi* parasites during asexual development and RNA decay analysis.

**Description:** The raw and annotated outputs from DEseq2 of transcript abundance in 3D7 and *Δpfdozi* parasites at 10, 20, 30 and 40 h post infection, respectively (Table S1A-F). Raw reads of RNA-seq for RNA decay studies in 3D7 and *Δpfdozi* clone K6. RNA was isolated at 0, 5, 15, 45, 60, 180 and 300 min after actinomycin D treatment (Table S1G), related to Fig. 4, S7.

**Supplementary data 2**

**Title:** RNA-seq and GO analysis of 3D7 and *Δpfdozi* parasites at the gametocyte stage.

**Description:** The raw and annotated outputs from DEseq2 of transcript abundance in 3D7 and *Δpfdozi* parasites at the gametocyte stage (Table S2A). GO analysis of downregulated (Table S2B) or upregulated (Table S2C) transcripts in the *Δpfdozi* parasites at the gametocyte stage, related to Fig. 5.

**Supplementary data 3**

**Title:** RIP-seq analysis of PfDOZI in asexual and sexual stages.

**Description:** PfDOZI target mRNAs in schizonts (Table S3A-B) and gametocytes (Table S3C), related to Fig. 6.

**Supplementary data 4**

**Title:** The PfDOZI interactomes in asexual and sexual stages.

**Description:** Full list of detected proteins and MPMP analysis in PfDOZI::GFP schizonts (Table S4A-B), gametocytes (Table S4C-D) and stressed schizonts (Table S4E-F). Full list of detected proteins in reciprocal pulldowns with PfDCP2::GFP (Table S4I-J) and PfGBP2::GFP (Table S4K-L), related to Fig. 7.

**Supplementary data 5**

**Title:** RNA-seq analysis of 3D7 and *Δpfdozi* parasites under nutrient stress.

**Description:** The raw and annotated outputs from DEseq2 comparison of transcript abundance in 3D7, *Δpfdozi*, stressed 3D7, and stressed *Δpfdozi* in the schizont stage, related to Fig. 8.

**Supplementary data 6**

**Title:** List of primers used in the study.

**Description:** The primers used in this study are shown here.
